# Supplementary material for: Associations between physical activity prior to infection and COVID-19 disease severity and symptoms: results from the prospective Predi-COVID cohort study
Source: BMJ Open. 2022 Apr 28;12(4):e057863. doi: 10.1136/bmjopen-2021-057863 (PMC9058293; doi:10.1136/bmjopen-2021-057863)
Supplement: Author's manuscript [file bmjopen-2021-057863.draft_revisions.pdf]

BMJ Open

BMJ Open is committed to open peer review. As part of this commitment we make the peer review history of every article we publish publicly available.

When an article is published we post the peer reviewers' comments and the authors' responses online. We also post the versions of the paper that were used during peer review. These are the versions that the peer review comments apply to.

The versions of the paper that follow are the versions that were submitted during the peer review process. They are not the versions of record or the final published versions. They should not be cited or distributed as the published version of this manuscript.

BMJ Open is an open access journal and the full, final, typeset and author-corrected version of record of the manuscript is available on our site with no access controls, subscription charges or pay-per-view fees (http://bmjopen.bmj.com).

If you have any questions on BMJ Open's open peer review process please email info.bmjopen@bmj.com

BMJ Open

Associations between physical activity prior to infection and COVID-19 disease severity and symptoms: results from the prospective Predi-COVID cohort study

Journal:	*BMJ Open*
Manuscript ID	bmjopen-2021-057863
Article Type:	Original research
Date Submitted by the Author:	30-Sep-2021
Complete List of Authors:	Malisoux, Laurent; Luxembourg Institute of Health, Department of Population Health<br>Backes, Anne; Luxembourg Institute of Health, Department of Population Health<br>Fischer, Aurélie; Luxembourg Institute of Health, Departement of Population Health<br>Aguayo, Gloria; Luxembourg Institute of Health, Department of Population Health<br>Ollert, Markus; Luxembourg Institute of Health, Director of Department of Infection & Immunity; University of Southern Denmark, Department of Dermatology and Allergy Center<br>Fagherazzi, Guy; Luxembourg Institute of Health, Department of Population Health
Keywords:	COVID-19, EPIDEMIOLOGY, Public health < INFECTIOUS DISEASES, SPORTS MEDICINE

SCHOLARONE™
Manuscripts

I, the Submitting Author has the right to grant and does grant on behalf of all authors of the Work (as defined in the below author licence), an exclusive licence and/or a non-exclusive licence for contributions from authors who are: i) UK Crown employees; ii) where BMJ has agreed a CC-BY licence shall apply, and/or iii) in accordance with the terms applicable for US Federal Government officers or employees acting as part of their official duties; on a worldwide, perpetual, irrevocable, royalty-free basis to BMJ Publishing Group Ltd ("BMJ") its licensees and where the relevant Journal is co-owned by BMJ to the co-owners of the Journal, to publish the Work in this journal and any other BMJ products and to exploit all rights, as set out in our licence.

The Submitting Author accepts and understands that any supply made under these terms is made by BMJ to the Submitting Author unless you are acting as an employee on behalf of your employer or a postgraduate student of an affiliated institution which is paying any applicable article publishing charge ("APC") for Open Access articles. Where the Submitting Author wishes to make the Work available on an Open Access basis (and intends to pay the relevant APC), the terms of reuse of such Open Access shall be governed by a Creative Commons licence – details of these licences and which Creative Commons licence will apply to this Work are set out in our licence referred to above.

Other than as permitted in any relevant BMJ Author's Self Archiving Policies, I confirm this Work has not been accepted for publication elsewhere, is not being considered for publication elsewhere and does not duplicate material already published. I confirm all authors consent to publication of this Work and authorise the granting of this licence.

For peer review only

Associations between physical activity prior to infection and COVID-19 disease severity and symptoms: results from the prospective Predi-COVID cohort study

Laurent Malisoux, PhD[a]

Anne Backes, MSc[a]

Aurélie Fischer, MSc[b]

Gloria Aguayo, PhD[b]

Markus Ollert, PhD[c,d]

Guy Fagherazzi, PhD[b]

[a]Physical Activity, Sport and Health Research Group, Department of Population Health, Luxembourg Institute of Health, Luxembourg.

[b]Deep Digital Phenotyping Research Unit, Department of Population Health, Luxembourg Institute of Health, Luxembourg.

[c]Department of Infection and Immunity, Luxembourg Institute of Health, Esch-sur-Alzette, Luxembourg.

[d]Department of Dermatology and Allergy Center, Odense Research Center for Anaphylaxis, University of Southern Denmark, Odense, Denmark.

Corresponding author

Laurent Malisoux, Physical Activity, Sport and Health Research Group, Luxembourg Institute of Health, 76 rue d'Eich, L-1460 Luxembourg. E-Mail address: laurent.malisoux@lih.lu

Word count: 2998 words

For peer review only

ABSTRACT

Objective: To investigate if physical activity (PA) prior to infection is associated with the severity of the disease in patients positively tested with COVID-19, as well as with the most common symptoms.

Design: A cross-sectional study using baseline data from a prospective, hybrid cohort study (Predi-COVID) in Luxembourg. Data were collected from May 2020 to June 2021

Setting: Real-life setting (at home) and hospitalised patients.

Participants: All volunteers aged >18 years with confirmed SARS-CoV-2 infection, as determined by reverse transcription polymerase chain reaction, and having completed the PA questionnaire (n=452).

Primary and secondary outcome measures: The primary outcome was disease severity (asymptomatic, mild illness and moderate illness). The secondary outcomes were self-reported symptoms.

Results: From the 452 patients included, 216 (48%) were female, the median (interquartile range) age was 42 (31, 51) years, 59 (13%) were classified as asymptomatic, 287 (63%) as mild illness, and 106 (24%) as moderate illness. The most prevalent symptoms were fatigue (n=294; 65%), headache (n=281; 62%) and dry cough (n=241; 53%). After adjustment, the highest PA level was associated with a lower risk of moderate illness (Odds ratio – OR: 0.37; 95% Confidence Interval – CI: 0.14-0.98, *p*=.045), fatigue (OR: 0.54; 95% CI: 0.30-0.97, *p*=.040), dry cough (OR: 0.55; 95% CI: 0.32-0.96, *p*=.034), and chest pain (OR: 0.32; 95% CI: 0.14-0.77, *p*=.010).

Conclusions: PA before COVID-19 infection was associated with a reduced risk of moderate illness severity and a reduced risk of experiencing fatigue, dry cough and chest pain, suggesting that engaging in PA may be an effective approach to minimise the severity of COVID-19.

Trial registration: The Predi-COVID study was registered on www.clinicaltrials.gov (identifier: NCT04380987).

Keywords: SARS-CoV-2, epidemiology, coronavirus infection, protective factors, physical activity behaviour.

For peer review only

ARTICLE SUMMARY

Strengths and limitations of the study

- This is the first study to investigate the association between physical activity prior to infection and COVID-19 severity among people with mild and moderate courses in real-life settings.
- The study only includes adults with confirmed SARS-CoV-2 infection as determined by reverse transcription-polymerase chain reaction, and classified as asymptomatic, mild or moderate cases according to an adapted version of the National Institute of Health symptom severity classification scheme.
- One of the main limitations of this study is that physical activity in the year before infection was assessed using a self-reported e-questionnaire, yet it covered all the physical activity domains (i.e., occupational, transportation, leisure-time, household/gardening).
- Multinomial logistic regression models and seperated logistic regression models were performed to investigate the association between physical activity and disease severity or specific symptoms.
- An in-depth analysis was conducted by controlling the models for the most relevant confounding factors identified so far.

For peer review only - http://bmjopen.bmj.com/site/about/guidelines.xhtml

INTRODUCTION

Coronavirus Disease 2019 (COVID-19), caused by severe acute respiratory syndrome coronavirus 2 (SARS-CoV-2), spread rapidly from China, caused outbreaks in countries throughout the world and was characterized by the World Health Organization (WHO) as a global pandemic.[1] This pandemic overwhelmed healthcare facilities, including but not limited to hospitals, intensive care units (ICU) and outpatient facilities.[2] Epidemiological studies have demonstrated that mortality is higher among the elderly population and the incidence is lower among children.[3] The risk for serious disease and death related to COVID-19 have been shown to be associated with baseline characteristics of patients such as old age, obesity, heavy smoking, as well as underlying conditions or comorbidities such as autoimmunity[4], genetic errors of immunity[5], hypertension, respiratory disease and cardiovascular disease.[6]

Physical activity (PA) is one of the leading determinants of health[7], and thus, lack of PA may worsen the impact of the current pandemic. Indeed, the risk of developing chronic diseases is much higher in those with low PA[8, 9], while COVID-19 patients with such underlying medical conditions (e.g., obesity and diabetes) are more likely to be hospitalized and have a greater likelihood in poorer clinical outcomes.[10] It is also well established that insufficient levels of PA lead to reduced respiratory and cardiovascular capacities, which can lead to a greater occurrence of obesity and other chronic diseases.[11] Moreover, there is growing evidence that PA has a protective effect against infectivity and severity of respiratory infection due probably to a better immunological response.[12] Consequently, one may argue that both low PA, an important modifiable factor, and high chronic disease prevalence worsen the severity of the crisis we are currently facing.

To date, the heterogeneity in the response to the infection to SARS-CoV-2 remains largely unexplained. COVID-19 symptoms are very heterogeneous and can range from minimal to significant severity in an infected individual.[13] A systematic review including 152 studies and 41,409 individuals showed that the most common symptoms were fever (59%), cough (55%), dyspnoea (31%), malaise (30%), fatigue (28%), sore throat (14%), headache (12%), and chest pain (11%).[14] While it has been demonstrated that PA decreases

For peer review only

For peer review only - http://bmjopen.bmj.com/site/about/guidelines.xhtml

the risk of severe clinical COVID-19 outcomes (e.g. hospitalisation or death)[15, 16], there is still limited information on the impact of PA on the severity of COVID-19 in patients with less severe disease and on the risk of developing specific symptoms. Therefore, the primary objective of this study was to investigate if the level of PA prior to infection is associated with the severity of the disease in patients positively tested with COVID-19. The secondary objective was to investigate if PA is associated with the most common symptoms (e.g. fever, cough, fatigue, etc.). We hypothesised that higher level of PA prior to infection would be associated with less severe forms of COVID-19, as well as with less frequent reports of the major Covid-19 related symptoms.

METHODS

Study design and participants

This is a cross-sectional study using data from a prospective, hybrid cohort study (Predi-COVID) composed of people positively tested for COVID-19 in Luxembourg.[2] The Predi-COVID study aims to identify epidemiological, clinical and sociodemographic characteristics as well as pathogen and/or host predictive biomarkers for the severity of COVID-19. The full study protocol has been published previously [2], with some of the methods that are relevant to this study reproduced below. The study was approved by the National Research Ethics Committee of Luxembourg in April 2020 (study number 202003/07) and registered on www.clinicaltrials.gov (identifier: NCT04380987). All volunteers received a full description of the protocol and provided written informed consent for participation. The findings from this study have been reported according to the *Strengthening the Reporting of Observational Studies in Epidemiology (STROBE)* statement.[17]

All individuals positively tested for COVID-19 in Luxembourg were eligible for the study and contacted by phone by the Health Inspection to enquire whether they consent to having their contact details communicated to the research team. The recruitment took place between Mai 2020 and June 2021. Inclusion criteria for this study were: having signed the informed consent, aged above 18 years, confirmed SARS-CoV-2 infection as determined by reverse transcription polymerase chain reaction (RT-PCR), performed by one of

For peer review only - http://bmjopen.bmj.com/site/about/guidelines.xhtml

the certified laboratories in Luxembourg, and having completed the questionnaire on PA behaviour. Patients already included in another interventional study on COVID-19 and those unable to understand French or German were excluded from the study.

Patient and public involvement

No patient or public involved.

Outcomes

All clinical data were collected at baseline by research nurses using a modified version of the International Severe Acute Respiratory and Emerging Infection Consortium (ISARIC) case report form. The primary outcome was the severity of illness, which was classified using an adapted version of the National Institute of Health symptom severity classification scheme.[18] Participants were grouped into the following three categories: asymptomatic (positive RT-PCR test and no symptom), mild illness (positive RT-PCR test and one or more symptoms, but no shortness of breath, no symptoms of lower respiratory disease, no abnormal chest imaging) and moderate illness (positive RT-PCR test and symptoms of lower respiratory disease or abnormal chest imaging). The secondary outcomes were specific symptoms reported by the participants at baseline. The presence of the following twelve symptoms was considered for the present work: headache, sore throat, fever, dry cough, diarrhoea, breathing difficulties, loss of taste and smell, chest pain, muscle pain, fatigue, confusion and falls.

Exposures

The exposure was PA prior to infection, which was assessed using a self-reported e-questionnaire using the electronic Patient Reported Outcomes (ePRO) module of Ennov Clinical. The PA questionnaire included questions on weekly hours spent walking (to work, shopping, and leisure time), cycling (to work, shopping, and leisure time), gardening (and other handiwork), in household chores, and sports activities (e.g. racket sports, swimming, running) in the year prior to infection, each reported for winter and summer,

For peer review only

For peer review only - http://bmjopen.bmj.com/site/about/guidelines.xhtml

separately.[19] The time reported for the two seasons was first averaged. Then each activity was assigned a metabolic equivalent task (MET) value based on the Compendium of PA[20], which included MET values of 3.0 for walking and household, 4.0 for gardening and 6.0 for cycling and sports. A total weekly METs score (in MET-h/week) was then calculated from the self-reported data. In addition, PA was categorised into four according to METs score using quartiles.

Covariates

Potential confounders were considered in the analyses and collected with the ISARIC case report form. They included age (years), sex, body mass index (BMI), as well as self-reported comorbidities, smoking status, income and sedentary behaviour. BMI was calculated as measured weight (kg)/height^2 (m^2). Comorbidities included hypertension, chronic heart disease, chronic pulmonary disease, asthma, chronic kidney disease, chronic kidney insufficiency with dialysis, liver disease (mild disease), liver disease (moderate or severe disease), chronic neurological disorders, malignant neoplasia/cancer, chronic hematologic disease, acquired immunodeficiency syndrome, obesity, diabetes with complications, diabetes without complications, rheumatological disease, dementia, malnutrition and chronic obstructive pulmonary disease. As few participants experienced comorbidities, this variable was categorized into "no comorbidity" and "at least one comorbidity". Participants were asked to report whether they are "never smoker", "former smoker" and "current smoker". Income was categorized into "<3000 euro/month", "3000-4999 euro/month", "5000-10000 euro/month" and ">10000 euro/month". Sedentary behaviour was defined as self-reported average number of daily hours spent in sedentary behaviour (e.g. at work, during meal, in front of the screen, etc.) prior to infection.

Statistical analysis

Descriptive statistics of the study population are presented as counts and percentage for categorical variables and as median and interquartile range (IQR) for not normally distributed continuous variables.

For peer review only

For peer review only - http://bmjopen.bmj.com/site/about/guidelines.xhtml

Multiple imputation was performed to deal with missing data. A multivariate imputation by chained equation (MICE) approach was used, assuming a missing at random mechanism. The best predictors were selected based on correlation with the outcomes[21] using the *quickpred* function from the *MICE* package in R. Ten datasets with 20 iterations were imputed and the plausibility of imputations were checked with density plots and summaries. Each imputed dataset was used separately to build the statistical models. Coefficients were pooled and confidence intervals were calculated based on Rubin's rules.[22]

Multinomial logistic regression models were used to investigate the association between PA and illness severity. Two different models were fitted: i) unadjusted model (Model 1), and ii) model 1 adjusted for age, sex, BMI, comorbidities, smoking status, income and sedentary behaviour (Model 2). Separate logistic regression models (fully adjusted) were also used to investigate the association between PA and specific COVID-19 symptoms. For both outcomes, PA was considered as a continuous and a categorical variable in distinct models.

Cubic spline regression models were plotted to investigate the potential non-linear associations between PA and the risk of mild and moderate illness severity, compared to an asymptomatic form, as well as between PA and the risk of specific symptoms. Each cubic spline regression model was defined with four knots, placed at the tertiles of the PA distribution, and with a reference exposure value set at the median of PA for disease severity or a specific symptom, respectively. The *splines* R package was used to fit the models.

All the statistical analyses were performed in R (version 3.6.1) using RStudio (version 1.3.1093). Statistical significance was set to $p<0.05$.

RESULTS

The analysis includes 452 adults with confirmed SARS-CoV-2 infection who agreed to participate in the study and provided data on PA. Only five participants were hospitalised, but none of them was admitted to ICU. Thirteen percent of the participants were asymptomatic (n=59), 63% were classified as mild illness (n=287), and 24% as moderate illness (n=106). The most prevalent symptoms were fatigue (n=294; 65%), headache (n=281; 62%), dry cough (n=241; 53%), muscle pain (n=237; 52%), sore throat (n=203; 45%), fever (n=197;

For peer review only

44%) and loss of taste and smell (n=179; 40%). Breathing difficulties (n=101; 22%), diarrhoea (n=89; 20%), chest pain (n=69; 15%), confusion (n=51; 10%) and falls (n=2; <1%) were less common.

Descriptive statistics of the study population stratified by illness severity are presented in Table 1. Overall, the study population included 48% of women (n=216), median age was 42 (IQR: 31 to 51), BMI was 24.9 (IQR: 22.1 to 27.8), and 79% did not suffer from any comorbidity (n=359). Missing data varied from 0 to 5%. The variables that had missing data were income (n=21; 5%), sedentary behaviour (n=3; 0.66%), BMI (n=2; 0.44%), age (n=1; 0.22%), and smoking status (n=1; 0.22%).

For peer review only

For peer review only - http://bmjopen.bmj.com/site/about/guidelines.xhtml

200 **Table 1.** Descriptive statistics of the study population stratified by disease severity.

Characteristic	All (n=452) *MED* (*IQR*) or *n* (%)	Disease severity		
		Asymptomatic (n=59) *MED* (*IQR*) or *n* (%)	Mild illness (n=287) *MED* (*IQR*) or *n* (%)	Moderate illness (n=106) *MED* (*IQR*) or *n* (%)
Age (years)†	42 (31, 51)	43 (31, 56)	41 (31, 51)	42 (32, 49)
Sex				
Female	216 (47.8)	19 (32.2)	134 (46.7)	63 (59.4)
Male	236 (52.2)	40 (67.8)	153 (53.3)	43 (40.6)
BMI (kg/m^2)†	24.9 (22.1, 27.8)	25.5 (22.2, 28.2)	24.7 (22.1, 27.5)	25.5 (22.2, 29.2)
Comorbidities				
No comorbidities	359 (79.4)	42 (71.2)	243 (84.7)	74 (69.8)
At least one comorbidity	93 (20.6)	17 (28.8)	44 (15.3)	32 (30.2)
Smoking status†				
Never smoker	291 (64.4)	34 (57.6)	184 (64.1)	73 (68.9)
Former smoker	84 (18.6)	13 (22.0)	55 (19.2)	16 (15.1)
Current smoker	77 (17.0)	12 (20.3)	48 (16.7)	17 (16.0)
Income (euro/month)†				
< 3000	71 (15.7)	11 (18.6)	39 (13.6)	21 (19.8)
3000-4999	110 (24.3)	15 (25.4)	70 (24.4)	25 (23.6)
5000-10000	203 (44.9)	23 (39.0)	138 (48.1)	42 (39.6)
> 10000	68 (15.0)	11 (18.6)	40 (13.9)	18 (17.0)
Sedentary behaviour (h/day)†	7 (4, 10)	6 (4, 10)	7 (4, 10)	6 (4, 9)
Physical activity (MET-h/week)	52.9 (30.8, 82.3)	63.0 (40.3, 98.5)	52.00 (31.4, 81.0)	49.3 (27.4, 73.9)
Physical activity (MET-h/week)				
< 30	108 (23.9)	10 (16.9)	68 (23.7)	30 (28.3)
30-52	113 (25.0)	13 (22.0)	75 (26.1)	25 (23.6)
52-82	116 (25.7)	16 (27.1)	74 (25.8)	26 (24.5)
> 82	115 (25.4)	20 (33.9)	70 (24.4)	25 (23.6)

201 Abbreviations: BMI = body mass index; IQR = interquartile range; MED = median; MET = metabolic equivalent task.

202 † Imputed data was used for the descriptive statistics.

For peer review only

Table 2 presents the unadjusted and adjusted models for the association between PA and disease severity. When PA was considered as a continuous variable, no association was found with mild or moderate forms of COVID-19 in the unadjusted model. After adjustment, greater PA was associated with a slightly lower risk of moderate illness (odd ratio - OR [95% confidence interval - CI]: 0.99 [0.98; 1.00], *P*=0.041). Cubic spline regression analysis showed that the relationship between PA and the risk of mild or moderate illness was not linear (Figure 1), which supports the use of PA as a categorical variable. The unadjusted model did not reveal any association between PA categories and mild or moderate illness. However, the adjusted model showed a lower risk of moderate illness in the category with the highest PA level (OR [95% CI]: 0.37 [0.14; 0.98], *P*=0.045).

Table 2. Associations between physical activity and illness severity.

Exposure	Outcome	Model 1		Model 2	
		**OR (95% CI)**	***p*-value**	**OR (95% CI)**	***p*-value**
PA (MET-h/week)	Disease severity†				
	Mild illness	0.99 (0.99, 1.00)	0.106	0.99 (0.99, 1.00)	0.064
	Moderate illness	0.99 (0.99, 1.00)	0.068	0.99 (0.98, 1.00)	**0.041***
PA (MET-h/week)††	Disease severity†				
30-52	Mild illness	0.85 (0.35, 2.06)	0.717	0.75 (0.30, 1.88)	0.542
52-82	Mild illness	0.68 (0.29, 1.60)	0.378	0.55 (0.22, 1.34)	0.185
> 82	Mild illness	0.51 (0.22, 1.18)	0.117	0.46 (0.19, 1.08)	0.075
30-52	Moderate illness	0.64 (0.24, 1.71)	0.374	0.57 (0.20, 1.58)	0.278
52-82	Moderate illness	0.54 (0.21, 1.40)	0.205	0.48 (0.18, 1.29)	0.145
> 82	Moderate illness	0.42 (0.16, 1.05)	0.064	0.37 (0.14, 0.98)	**0.045***
p-trend	Mild illness	0.99 (0.98, 1.01)	0.374	0.99 (0.97, 1.01)	0.243
p-trend	Moderate illness	0.99 (0.97, 1.01)	0.203	0.99 (0.97, 1.01)	0.171

Values are presented as OR (95% CI), which were calculated according to Rubin's rule. All models were performed with imputed data. Model 1 = unadjusted model; Model 2 = model 1 adjusted for age, sex, BMI, comorbidities, smoking status, income and sedentary behaviour. Abbreviations: CI = confidence interval; MET = metabolic equivalent task; OR = odds ratio; PA = physical activity.
† Reference: asymptomatic; †† Reference: < 30 MET-h/week; * p-value < 0.05.

Insert Figure 1 about here

For peer review only

The associations between PA and specific symptoms in the adjusted models are presented in Table 3. Greater PA was associated with lower risk of chest pain (OR [95% CI]: 0.99 [0.98; 1.00], *P*=0.007) when PA was considered as a continuous variable. The category with the highest PA level was associated with lower risk of fatigue (OR [95% CI]: 0.54 [0.30; 0.97], *P*=0.040), dry cough (OR [95% CI]: 0.55 [0.32; 0.96], *P*=0.034), and chest pain (OR [95% CI]: 0.32 [0.14; 0.77], *P*=0.010). Figure 2 shows separate cubic splines investigating the association between PA and specific COVID-19 symptoms.

Table 3. Associations between physical activity and specific COVID-19 symptoms using the adjusted model 2.

**Exposure**	**Outcome**	**OR (95% CI)**	***p*-value**
PA (MET-h/week)	Symptom		
	Fatigue	1.00 (0.99, 1.00)	0.130
	Headache	1.00 (0.99, 1.00)	0.181
	Muscle pain	1.00 (0.99, 1.00)	0.442
	Dry cough	1.00 (0.99, 1.00)	0.056
	Sore throat	1.00 (1.00, 1.00)	0.973
	Fever	1.00 (0.99, 1.00)	0.453
	Loss of taste and smell	1.00 (0.99, 1.00)	0.286
	Breathing difficulties	1.00 (0.99, 1.00)	0.348
	Diarrhoea	1.00 (0.99, 1.00)	0.577
	Chest pain	0.99 (0.98, 1.00)	**0.007***
	Confusion	1.00 (0.99, 1.01)	0.804
PA (MET-h/week)†	Symptom		
30-52		0.63 (0.35, 1.15)	0.130
52-82	Fatigue	0.69 (0.37, 1.25)	0.218
> 82		0.54 (0.30, 0.97)	**0.040***
30-52		1.10 (0.62, 1.94)	0.745
52-82	Headache	1.07 (0.61, 1.89)	0.807
> 82		0.73 (0.42, 1.26)	0.256
30-52		0.80 (0.46, 1.39)	0.430
52-82	Muscle pain	1.15 (0.67, 1.99)	0.615
> 82		0.68 (0.40, 1.18)	0.171
30-52		0.66 (0.38, 1.16)	0.145
52-82	Dry cough	0.69 (0.40, 1.21)	0.196
> 82		0.55 (0.32, 0.96)	**0.034***
30-52		1.24 (0.71, 2.16)	0.453
52-82	Sore throat	1.34 (0.77, 2.33)	0.297
> 82		1.19 (0.69, 2.07)	0.531
30-52		0.97 (0.56, 1.69)	0.924
52-82	Fever	0.90 (0.52, 1.56)	0.700
> 82		0.89 (0.51, 1.54)	0.675
30-52		1.02 (0.58, 1.79)	0.947
52-82	Loss of taste and smell	1.09 (0.62, 1.90)	0.769
> 82		0.84 (0.47, 1.48)	0.539

For peer review only

30-52	Breathing difficulties	0.64 (0.33, 1.25)	0.190
52-82		0.86 (0.45, 1.62)	0.634
> 82		0.72 (0.38, 1.37)	0.318
30-52	Diarrhoea	0.72 (0.36, 1.43)	0.343
52-82		0.94 (0.49, 1.82)	0.862
> 82		0.70 (0.36, 1.39)	0.313
30-52	Chest pain	0.80 (0.39, 1.65)	0.553
52-82		0.87 (0.43, 1.78)	0.705
> 82		0.32 (0.14, 0.77)	**0.010***
30-52	Confusion	1.04 (0.40, 2.69)	0.941
52-82		2.16 (0.91, 5.09)	0.079
> 82		0.99 (0.38, 2.61)	0.982

Values are presented as OR (95% CI), which were calculated according to Rubin's rule. All models were performed with imputed data. Abbreviations: CI = confidence interval; MET = metabolic equivalent task; OR = odds ratio; PA = physical activity.

† Reference: < 30 MET-h/week; * p-value < 0.05.

Insert Figure 2 about here

DISCUSSION

The protective effect of meeting PA recommendations on the risk of severe COVID-19 outcomes (i.e., death, ICU admission and hospitalisation) has previously been documented.[15, 16] However, whether PA may also prevent less severe illness courses remains unknown. The primary objective of this study was to investigate if PA is associated with illness severity among patients with asymptomatic, mild or moderate COVID-19 severity. The secondary objective was to investigate if PA is associated with the most common symptoms such as fatigue, headache, dry cough, muscle pain, sore throat, fever and loss of taste and smell. We hypothesised that higher level of PA prior to the infection would be associated with lower risk of mild and moderate illness, and lower risk of suffering from some of the most commonly reported symptoms. Our main findings were that participants with greater PA were at a lower risk of moderate COVID-19 severity, which confirms our hypothesis. Furthermore, greater level of PA was also associated with a decreased risk of experiencing fatigue, dry cough and chest pain, which are among the most commonly reported symptoms in patients positively tested with COVID-19. These findings suggest that PA is a protective factor for the development of moderate COVID-19 course in adults and for some common related symptoms.

For peer review only - http://bmjopen.bmj.com/site/about/guidelines.xhtml

Previous studies have shown that insufficient PA prior to the pandemic increased the risk of hospitalization[15, 16, 23-25], admission to ICU and death.[15, 16] Notably, low PA was shown to be one of the stronger risk factors for severe COVID-19 outcome, after advanced age and history of organ transplant.[16] Furthermore, meeting the PA guidelines[26] has been shown to decrease the risk of SARS-CoV-2 infection in adult Koreans, beside the negative association with the risk of severe COVID-19 illness (ICU admission or administration of invasive ventilation) and COVID-19 related death.[15] Objective measures of PA have also demonstrated a decreased risk of contracting COVID-19 and hospitalisation in those with greater PA.[27] A study including only patients with chest computed tomography scan confirming infection showed that physical inactivity was associated with the severity of COVID-19 disease.[28] Overall, these previous studies suggested a protective effect of PA for severe COVID-19 outcomes, while some of these studies only included severe cases. While our findings are in line with these previous observations as they confirm the benefits of PA for COVID-19 severity, this is the first study to demonstrate that PA can also provide a protective effect for moderate courses.

Previous scientific literature has supported the role of PA against upper respiratory tract infections.[29] Research on the 2009 H1N1 influenza epidemic demonstrated a dose-response relationship between PA performed before infection and a reduction in the incidence, duration, or severity of acute upper respiratory tract infections.[30] During seasonal influenza, moderately active and active individuals were approximately 15% less likely to visit a physician or emergency services due to influenza compared to inactive individuals.[31] A recent meta-analysis revealed that people engaged in higher levels of PA showed a 31% risk reduction for community acquired infectious disease.[12] PA can play a protective role against respiratory viral infections through increasing the endurance and strength of the respiratory muscles and improving the immune response to respiratory viral antigens.[32] Regular exercise induces improvements in respiratory, cardiovascular and metabolic adaptations and results in higher maximum oxygen uptakes, breathing frequency, stroke volume, and cardiac output to name a few.[33] The improvement in

For peer review only - http://bmjopen.bmj.com/site/about/guidelines.xhtml

cardiometabolic and respiratory function help boost the immune defence system.[34] Consistently, maximal exercise capacity prior SARS-CoV-2 infection was shown to be inversely associated with the risk of hospitalization due to COVID-19.[35]

The immune system is very responsive to PA and exercise, with the extent and duration depending on the degree of physiological stress imposed by the workload. The beneficial effect of regular PA on the immune system may involve several mechanisms such as enhanced immunosurveillance, reduced systemic inflammation and improved regulation of the immune system as well as delayed onset of immunosenescence.[34, 36] Each session of moderate intensity PA stimulates an increase in the antipathogen activity of immune system macrophages as well as in the recirculation of key immune system cells, immunoglobulins and anti-inflammatory cytokines in the blood. Interestingly, PA may also enhance vaccination response.[37]

Some studies have described a "J" shaped association between exercise volume and infection with optimal protection at moderate levels of activity.[38] In this study, the cubic spline plots showed that the relationships between PA and COVID-19 severity and symptoms occurrence were not linear, but our sample size did not enable to define the shape of the curve accurately.

One of the limitations of this study was the use of self-reported measures to assess PA, which might have resulted in recall bias, compared with exposure assessment measured using objective means (i.e., accelerometers), which can provide a more accurate assessment of the true level of PA. However, our PA assessment tool has previously been used in large cohort studies[19, 39], and covers all the PA domains (i.e., occupational, transportation, leisure-time, household/gardening). Nevertheless, the use of self-report questionnaires usually leads to overestimation of PA, which may lead to underestimation of the magnitude of true associations.[40] Second, there was no measure of PA intensity, although each activity was assigned a specific MET value. Third, this study was an observational study with a limited sample size for some

For peer review only

outcome categories. It is not possible to conclude that PA prior to infection is causally related to less severe COVID-19 outcomes as this study design suffers from a potential issue of residual confounding due to unmeasured or unknown confounders. However, our adjusted model controlled for all the most relevant confounders identified so far. Fourth, some estimated 95% CI suggest sparse data bias (see Table 2 and 3), which should be recognised as an important limitation.

CONCLUSION

We found that greater PA prior to infection was associated with a reduced risk of moderate illness severity among adults positively tested for COVID-19. Greater PA was also associated with a reduced risk of experiencing fatigue, dry cough and chest pain, which are among the most commonly reported symptoms in patients with COVID-19. This study provides new evidence that PA is a modifiable risk factor for COVID-19 severity, including moderate illness. Our findings suggest that engaging in regular PA may be one of the key actions individuals can take to minimise adverse consequences of COVID-19.

AUTHOR CONTRIBUTIONS

LM, AB, AF, GA, MO, GF contributed sufficiently to the manuscript to justify authorship. LM, AF, GA, MO and GF conceptualised the project, and LM, AB, GA and GF defined the methodology for the present study. LM and AB verified the underlying data and conducted the data analysis. All authors were involved in the interpretation of the analysis results. LM drafted the first manuscript and all other authors provided significant feedback and comments to refine the final manuscript. All authors approved the final manuscript and confirm that they accept responsibility to submit for publication.

ACKNOWLEDGEMENTS

We thank the Predi-COVID participants for their involvement in the study, the members of the Predi-COVID external scientific committee for their expertise, as well as the project team, the IT team in charge of the app

For peer review only

development and the nurses in charge of recruitment, data and sample collection and management on the field.

FUNDING/SUPPORT

The Predi-COVID study is supported by the Luxembourg National Research Fund (FNR) (Predi-COVID, grant number 14716273), the André Losch Fondation and the Luxembourg Institute of Health. The study funders had no role in the design and conduct of the study; collection, management, analysis, and interpretation of the data; preparation, review, or approval of the manuscript; or decision to submit the manuscript for publication.

COMPETING INTERESTS

The authors did not have any competing interest.

DATA SHARING STATEMENT

As this is a cross-sectional analysis of baseline data from a currently ongoing prospective study, the data will not be made available publicly before the end of the Predi-Covid study. The study protocol can be found under https://bmjopen.bmj.com/content/10/11/e041834.abstract.

ETHICS STATEMENT

The study was approved by the National Research Ethics Committee of Luxembourg (CNER) in April 2020 (ID: 202003/07).

For peer review only

REFERENCES

1. Liu YC, Kuo RL, Shih SR. COVID-19: The first documented coronavirus pandemic in history. *Biomed J* 2020;43(4):328-33.

2. Fagherazzi G, Fischer A, Betsou F, et al. Protocol for a prospective, longitudinal cohort of people with COVID-19 and their household members to study factors associated with disease severity: The Predi-COVID study. *BMJ Open* 2020;10(11):e041834.

3. Zhou F, Yu T, Du R, et al. Clinical course and risk factors for mortality of adult inpatients with COVID-19 in Wuhan, China: A retrospective cohort study. *Lancet* 2020;395(10229):1054-62.

4. Bastard P, Gervais A, Le Voyer T, et al. Autoantibodies neutralizing type I IFNs are present in~ 4% of uninfected individuals over 70 years old and account for~ 20% of COVID-19 deaths. *Science Immunology* 2021;6(62):eabl4340.

5. Asano T, Boisson B, Onodi F, et al. X-linked recessive TLR7 deficiency in~ 1% of men under 60 years old with life-threatening COVID-19. *Science Immunology* 2021;6(62):eabl4348.

6. Jordan RE, Adab P, Cheng K. Covid-19: Risk factors for severe disease and death. British Medical Journal Publishing Group; 2020.

7. Lee IM, Shiroma EJ, Lobelo F, et al. Effect of physical inactivity on major non-communicable diseases worldwide: An analysis of burden of disease and life expectancy. *Lancet* 2012;380(9838):219-29.

8. Lacombe J, Armstrong MEG, Wright FL, et al. The impact of physical activity and an additional behavioural risk factor on cardiovascular disease, cancer and all-cause mortality: A systematic review. *BMC Public Health* 2019;19(1):900.

9. Wahid A, Manek N, Nichols M, et al. Quantifying the association between physical activity and cardiovascular disease and diabetes: A systematic review and meta-analysis. *J Am Heart Assoc* 2016;5(9):e002495.

10. Bialek S, Boundy E, Bowen V, et al. Severe outcomes among patients with coronavirus disease 2019 (COVID-19)—United States, February 12–March 16, 2020. *Morb Mortal Wkly Rep* 2020;69(12):343.

11. Lavie CJ, Ozemek C, Carbone S, et al. Sedentary behavior, exercise, and cardiovascular health. *Circ Res* 2019;124(5):799-815.

12. Chastin SF, Abaraogu U, Bourgois JG, et al. Effects of regular physical activity on the immune system, vaccination and risk of community-acquired infectious disease in the general population: Systematic review and meta-analysis. *Sports Med* 2021:1-14.

13. Yuki K, Fujiogi M, Koutsogiannaki S. COVID-19 pathophysiology: A review. *Clin Immunol* 2020;215:108427.

14. da Rosa Mesquita R, Francelino Silva Junior LC, Santos Santana FM, et al. Clinical manifestations of COVID-19 in the general population: Systematic review. *Wien Klin Wochenschr* 2021;133(7-8):377-82.

15. Lee SW, Lee J, Moon SY, et al. Physical activity and the risk of SARS-CoV-2 infection, severe COVID-19 illness and COVID-19 related mortality in South Korea: A nationwide cohort study. *Br J Sports Med* 2021.

16. Sallis R, Young DR, Tartof SY, et al. Physical inactivity is associated with a higher risk for severe COVID-19 outcomes: A study in 48 440 adult patients. *Br J Sports Med* 2021.

17. von Elm E, Altman DG, Egger M, et al. The Strengthening the Reporting of Observational Studies in Epidemiology (STROBE) statement: Guidelines for reporting observational studies. *PLoS Med* 2007;4(10):e296.

For peer review only - http://bmjopen.bmj.com/site/about/guidelines.xhtml

18. National Institutes of Health (NIH). Clinical spectrum of SARS-CoV-2 infection 2021. https://www.covid19treatmentguidelines.nih.gov/overview/clinical-spectrum/ (accessed July 23, 2021).

19. MacDonald CJ, Madika AL, Lajous M, et al. Associations between physical activity and incident hypertension across strata of body mass index: A prospective investigation in a large cohort of french women. *J Am Heart Assoc* 2020;9(23):e015121.

20. Ainsworth BE, Haskell WL, Whitt MC, et al. Compendium of physical activities: an update of activity codes and MET intensities. *Med Sci Sports Exerc* 2000;32(9 Suppl):S498-504.

21. Van Buuren S, Groothuis-Oudshoorn K. mice: Multivariate imputation by chained equations in R. *J Stat Softw* 2011;45(1):1-67.

22. Rubin DB. Multiple imputation for nonresponse in surveys: John Wiley & Sons 2004.

23. Hamer M, Kivimaki M, Gale CR, et al. Lifestyle risk factors, inflammatory mechanisms, and COVID-19 hospitalization: A community-based cohort study of 387,109 adults in UK. *Brain Behav Immun* 2020;87:184-87.

24. Yuan Q, Huang H-y, Chen X-l, et al. Does pre-existent physical inactivity have a role in the severity of COVID-19? *Ther Adv Respir Dis* 2021;15:17534666211025221.

25. de Souza FR, Motta-Santos D, Dos Santos Soares D, et al. Association of physical activity levels and the prevalence of COVID-19-associated hospitalization. *J Sci Med Sport* 2021;24(9):913-18.

26. Piercy KL, Troiano RP, Ballard RM, et al. The Physical Activity Guidelines for Americans. *JAMA* 2018;320(19):2020-28.

27. Zhang X, Li X, Sun Z, et al. Physical activity and COVID-19: An observational and Mendelian randomisation study. *J Glob Health* 2020;10(2):020514.

28. Tavakol Z, Ghannadi S, Tabesh MR, et al. Relationship between physical activity, healthy lifestyle and COVID-19 disease severity: A cross-sectional study. *J Public Health* 2021:1-9.

29. Laddu DR, Lavie CJ, Phillips SA, et al. Physical activity for immunity protection: Inoculating populations with healthy living medicine in preparation for the next pandemic. *Prog Cardiovasc Dis* 2021;64:102-04.

30. Wong CM, Lai HK, Ou CQ, et al. Is exercise protective against influenza-associated mortality? *PLoS One* 2008;3(5):e2108.

31. Siu E, Campitelli MA, Kwong JC. Physical activity and influenza-coded outpatient visits: A population-based cohort study. *PLoS One* 2012;7(6):e39518.

32. Jakobsson J, Malm C, Furberg M, et al. Physical activity during the coronavirus (COVID-19) pandemic: Prevention of a decline in metabolic and immunological functions. *Front Sports Act Living* 2020;2:57.

33. Alkhatib A. Antiviral functional foods and exercise lifestyle prevention of coronavirus. *Nutrients* 2020;12(9):2633.

34. Nieman DC, Wentz LM. The compelling link between physical activity and the body's defense system. *J Sport Health Sci* 2019;8(3):201-17.

35. Brawner CA, Ehrman JK, Bole S, et al., editors. Inverse relationship of maximal exercise capacity to hospitalization secondary to coronavirus disease 2019. Mayo Clinic Proceedings; 2021: Elsevier.

36. Simpson RJ, Kunz H, Agha N, et al. Exercise and the regulation of immune functions. *Prog Mol Biol Transl Sci* 2015;135:355-80.

37. Pascoe AR, Fiatarone Singh MA, Edwards KM. The effects of exercise on vaccination responses: A review of chronic and acute exercise interventions in humans. *Brain Behav Immun* 2014;39:33-41.

For peer review only

38. Schwellnus M, Soligard T, Alonso J-M, et al. How much is too much?(Part 2) International Olympic Committee consensus statement on load in sport and risk of illness. *Br J Sports Med* 2016;50(17):1043-52.

39. Fournier A, Dos Santos G, Guillas G, et al. Recent recreational physical activity and breast cancer risk in postmenopausal women in the E3N cohort. *Cancer Epidemiol Biomarkers Prev* 2014;23(9):1893-902.

40. Stamatakis E, Lee I-M, Bennie J, et al. Does strength-promoting exercise confer unique health benefits? A pooled analysis of data on 11 population cohorts with all-cause, cancer, and cardiovascular mortality endpoints. *Am J Epidemiol* 2018;187(5):1102-12.

For peer review only

FIGURES

Figure 1.

Figure title: Cubic spline regression investigating the association between physical activity and disease severity. Reference exposure value set at the median of physical activity (52.9 MET-h/week).

Figure 2.

Figure title: Cubic spline regression investigating the association between physical activity and specific COVID-19 symptoms. Reference exposure value set at the median of physical activity (52.9 MET-h/week).

For peer review only

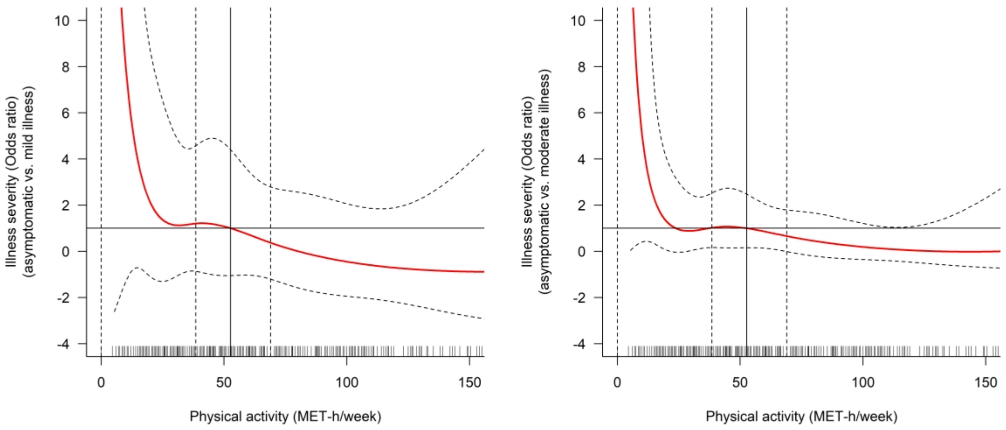

Cubic spline regression investigating the association between physical activity and disease severity. Reference exposure value set at the median of physical activity (52.9 MET-h/week).

762x317mm (236 x 236 DPI)

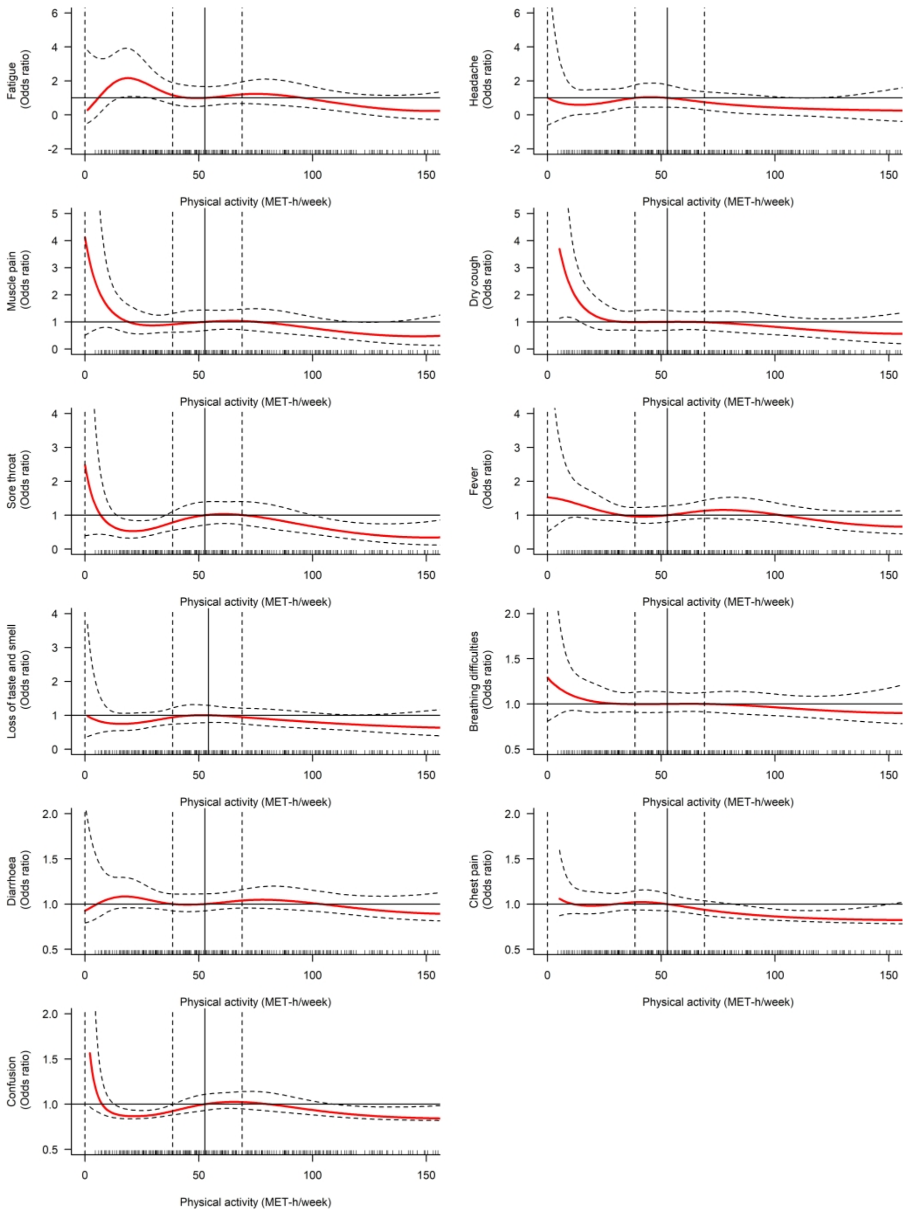

Cubic spline regression investigating the association between physical activity and specific COVID-19 symptoms. Reference exposure value set at the median of physical activity (52.9 MET-h/week).

571x762mm (236 x 236 DPI)

STROBE Statement—Checklist of items that should be included in reports of ***cross-sectional studies***

	Item No	Recommendation	Pages
**Title and abstract**	1	(*a*) Indicate the study's design with a commonly used term in the title or the abstract	2
		(*b*) Provide in the abstract an informative and balanced summary of what was done and what was found	2
**Introduction**			
Background/rationale	2	Explain the scientific background and rationale for the investigation being reported	4-5
Objectives	3	State specific objectives, including any prespecified hypotheses	5
**Methods**			
Study design	4	Present key elements of study design early in the paper	5
Setting	5	Describe the setting, locations, and relevant dates, including periods of recruitment, exposure, follow-up, and data collection	5-6
Participants	6	(*a*) Give the eligibility criteria, and the sources and methods of selection of participants	5-6
Variables	7	Clearly define all outcomes, exposures, predictors, potential confounders, and effect modifiers. Give diagnostic criteria, if applicable	6-7
Data sources/ measurement	8*	For each variable of interest, give sources of data and details of methods of assessment (measurement). Describe comparability of assessment methods if there is more than one group	6-7
Bias	9	Describe any efforts to address potential sources of bias	7-8
Study size	10	Explain how the study size was arrived at	5 and 8
Quantitative variables	11	Explain how quantitative variables were handled in the analyses. If applicable, describe which groupings were chosen and why	6-7
Statistical methods	12	(*a*) Describe all statistical methods, including those used to control for confounding	7-8
		(*b*) Describe any methods used to examine subgroups and interactions	n.a.
		(*c*) Explain how missing data were addressed	7-8
		(*d*) If applicable, describe analytical methods taking account of sampling strategy	n.a.
		(*e*) Describe any sensitivity analyses	n.a.
**Results**			
Participants	13*	(a) Report numbers of individuals at each stage of study—eg numbers potentially eligible, examined for eligibility, confirmed eligible, included in the study, completing follow-up, and analysed	8-9
		(b) Give reasons for non-participation at each stage	n.a.
		(c) Consider use of a flow diagram	n.a.

For peer review only

Descriptive data	14*	(a) Give characteristics of study participants (eg demographic, clinical, social) and information on exposures and potential confounders	8-9 (table 1)
		(b) Indicate number of participants with missing data for each variable of interest	9
Outcome data	15*	Report numbers of outcome events or summary measures	8-9
Main results	16	(*a*) Give unadjusted estimates and, if applicable, confounder-adjusted estimates and their precision (eg, 95% confidence interval). Make clear which confounders were adjusted for and why they were included	11-12 (tables 2 & 3)
		(*b*) Report category boundaries when continuous variables were categorized	7 (tables 2 & 3)
		(*c*) If relevant, consider translating estimates of relative risk into absolute risk for a meaningful time period	n.a.
Other analyses	17	Report other analyses done—eg analyses of subgroups and interactions, and sensitivity analyses	n.a.
**Discussion**			
Key results	18	Summarise key results with reference to study objectives	13
Limitations	19	Discuss limitations of the study, taking into account sources of potential bias or imprecision. Discuss both direction and magnitude of any potential bias	15-16
Interpretation	20	Give a cautious overall interpretation of results considering objectives, limitations, multiplicity of analyses, results from similar studies, and other relevant evidence	14-15
Generalisability	21	Discuss the generalisability (external validity) of the study results	13-14, 16
**Other information**			
Funding	22	Give the source of funding and the role of the funders for the present study and, if applicable, for the original study on which the present article is based	17

*Give information separately for exposed and unexposed groups.

Note: An Explanation and Elaboration article discusses each checklist item and gives methodological background and published examples of transparent reporting. The STROBE checklist is best used in conjunction with this article (freely available on the Web sites of PLoS Medicine at http://www.plosmedicine.org/, Annals of Internal Medicine at http://www.annals.org/, and Epidemiology at http://www.epidem.com/). Information on the STROBE Initiative is available at www.strobe-statement.org.

For peer review only

BMJ Open

Associations between physical activity prior to infection and COVID-19 disease severity and symptoms: results from the prospective Predi-COVID cohort study

Journal:	*BMJ Open*
Manuscript ID	bmjopen-2021-057863.R1
Article Type:	Original research
Date Submitted by the Author:	01-Mar-2022
Complete List of Authors:	Malisoux, Laurent; Luxembourg Institute of Health, Department of Precision Health<br>Backes, Anne; Luxembourg Institute of Health, Department of Precision Health<br>Fischer, Aurélie; Luxembourg Institute of Health, Department of Precision Health<br>Aguayo, Gloria; Luxembourg Institute of Health, Department of Precision Health<br>Ollert, Markus; Luxembourg Institute of Health, Director of Department of Infection & Immunity; University of Southern Denmark, Department of Dermatology and Allergy Center<br>Fagherazzi, Guy; Luxembourg Institute of Health, Department of Precision Health
**Primary Subject Heading**:	Epidemiology
Secondary Subject Heading:	Infectious diseases, Sports and exercise medicine
Keywords:	EPIDEMIOLOGY, Public health < INFECTIOUS DISEASES, SPORTS MEDICINE, COVID-19

SCHOLARONE™ Manuscripts

I, the Submitting Author has the right to grant and does grant on behalf of all authors of the Work (as defined in the below author licence), an exclusive licence and/or a non-exclusive licence for contributions from authors who are: i) UK Crown employees; ii) where BMJ has agreed a CC-BY licence shall apply, and/or iii) in accordance with the terms applicable for US Federal Government officers or employees acting as part of their official duties; on a worldwide, perpetual, irrevocable, royalty-free basis to BMJ Publishing Group Ltd ("BMJ") its licensees and where the relevant Journal is co-owned by BMJ to the co-owners of the Journal, to publish the Work in this journal and any other BMJ products and to exploit all rights, as set out in our licence.

The Submitting Author accepts and understands that any supply made under these terms is made by BMJ to the Submitting Author unless you are acting as an employee on behalf of your employer or a postgraduate student of an affiliated institution which is paying any applicable article publishing charge ("APC") for Open Access articles. Where the Submitting Author wishes to make the Work available on an Open Access basis (and intends to pay the relevant APC), the terms of reuse of such Open Access shall be governed by a Creative Commons licence – details of these licences and which Creative Commons licence will apply to this Work are set out in our licence referred to above.

Other than as permitted in any relevant BMJ Author's Self Archiving Policies, I confirm this Work has not been accepted for publication elsewhere, is not being considered for publication elsewhere and does not duplicate material already published. I confirm all authors consent to publication of this Work and authorise the granting of this licence.

For peer review only

Associations between physical activity prior to infection and COVID-19 disease severity and symptoms: results from the prospective Predi-COVID cohort study

Laurent Malisoux, PhD[a]

Anne Backes, MSc[a]

Aurélie Fischer, MSc[b]

Gloria A. Aguayo, MD, PhD[b]

Markus Ollert, MD, DMSci[c,d]

Guy Fagherazzi, PhD[b]

[a]Physical Activity, Sport and Health Research Group, Department of Precision Health, Luxembourg Institute of Health, Luxembourg.

[b]Deep Digital Phenotyping Research Unit, Department of Precision Health, Luxembourg Institute of Health, Luxembourg.

[c]Department of Infection and Immunity, Luxembourg Institute of Health, Esch-sur-Alzette, Luxembourg.

[d]Department of Dermatology and Allergy Center, Odense Research Center for Anaphylaxis, University of Southern Denmark, Odense, Denmark.

Corresponding author

Laurent Malisoux, Physical Activity, Sport and Health Research Group, Luxembourg Institute of Health, 76 rue d'Eich, L-1460 Luxembourg. E-Mail address: laurent.malisoux@lih.lu

Word count: 3311 words

For peer review only

ABSTRACT

Objective: To investigate if the physical activity (PA) prior to infection is associated with the severity of the disease in patients positively tested for COVID-19, as well as with the most common symptoms.

Design: A cross-sectional study using baseline data from a prospective, hybrid cohort study (Predi-COVID) in Luxembourg. Data were collected from May 2020 to June 2021

Setting: Real-life setting (at home) and hospitalised patients.

Participants: All volunteers aged >18 years with confirmed SARS-CoV-2 infection, as determined by reverse transcription-polymerase chain reaction, and having completed the PA questionnaire (n=452).

Primary and secondary outcome measures: The primary outcome was disease severity (asymptomatic, mild illness, and moderate illness). The secondary outcomes were self-reported symptoms.

Results: From the 452 patients included, 216 (48%) were female, the median (interquartile range) age was 42 (31, 51) years, 59 (13%) were classified as asymptomatic, 287 (63%) as mild illness, and 106 (24%) as moderate illness. The most prevalent symptoms were fatigue (n=294; 65%), headache (n=281; 62%) and dry cough (n=241; 53%). After adjustment, the highest PA level was associated with a lower risk of moderate illness (Odds ratio – OR: 0.37; 95% Confidence Interval – CI: 0.14-0.98, *p*=.045), fatigue (OR: 0.54; 95% CI: 0.30-0.97, *p*=.040), dry cough (OR: 0.55; 95% CI: 0.32-0.96, *p*=.034), and chest pain (OR: 0.32; 95% CI: 0.14-0.77, *p*=.010).

Conclusions: PA before COVID-19 infection was associated with a reduced risk of moderate illness severity and a reduced risk of experiencing fatigue, dry cough, and chest pain, suggesting that engaging in PA may be an effective approach to minimise the severity of COVID-19.

Trial registration: The Predi-COVID study was registered on www.clinicaltrials.gov (identifier: NCT04380987).

Keywords: SARS-CoV-2, epidemiology, coronavirus infection, protective factors, physical activity behaviour.

For peer review only

For peer review only - http://bmjopen.bmj.com/site/about/guidelines.xhtml

ARTICLE SUMMARY

Strengths and limitations of the study

- This is the first study to investigate the association between physical activity prior to infection and COVID-19 severity among people with mild and moderate courses in real-life settings.
- The study only includes adults with confirmed SARS-CoV-2 infection as determined by reverse transcription-polymerase chain reaction and classified as asymptomatic, mild, or moderate cases according to an adapted version of the National Institute of Health symptom severity classification scheme.
- One of the main limitations of this study is that physical activity in the year before infection was assessed using a self-reported e-questionnaire, yet it covered all the physical activity domains (i.e., occupational, transportation, leisure-time, household/gardening).
- Multinomial logistic regression models and separate logistic regression models were performed to investigate the association between physical activity and disease severity or specific symptoms.
- An in-depth analysis was conducted by controlling the models for the most relevant confounding factors identified so far.

For peer review only

For peer review only - http://bmjopen.bmj.com/site/about/guidelines.xhtml

INTRODUCTION

Coronavirus Disease 2019 (COVID-19), caused by severe acute respiratory syndrome coronavirus 2 (SARS-CoV-2), spread rapidly from China, caused outbreaks in countries throughout the world and was characterized by the World Health Organization (WHO) as a global pandemic on 11 March 2020.[1] This pandemic overwhelmed healthcare facilities, including but not limited to hospitals, intensive care units (ICU) and outpatient facilities.[2] Epidemiological studies have demonstrated that mortality is higher among the elderly population, with a 6.1% increase in mortality per 10 years increase in age.[3] The risk for serious disease and death related to COVID-19 have been shown to be associated with baseline characteristics of patients such as old age, obesity, heavy smoking, as well as underlying conditions or comorbidities such as autoimmunity[4], genetic errors of immunity[5], hypertension, respiratory disease and cardiovascular disease.[6]

Physical activity (PA) is one of the leading determinants of health[7], and thus, lack of PA may worsen the impact of the current pandemic. Indeed, the risk of developing chronic diseases is much higher in those with low PA[8, 9], while COVID-19 patients with such underlying medical conditions (e.g., obesity and diabetes) are more likely to be hospitalized and have a greater likelihood in poorer clinical outcomes.[10] It is also well established that insufficient levels of PA lead to reduced respiratory and cardiovascular capacities, which can lead to a greater occurrence of obesity and other chronic diseases.[11] Moreover, there is growing evidence that PA has a protective effect against infectivity and severity of respiratory infection due probably to a better immunological response.[12] Consequently, one may argue that both low PA, an important modifiable factor, and high chronic disease prevalence worsen the severity of the crisis we are currently facing.

To date, the heterogeneity in the response to the infection to SARS-CoV-2 remains largely unexplained. COVID-19 symptoms are very heterogeneous and can range from minimal to significant severity in an infected individual.[13] A systematic review including 152 studies and 41,409 individuals showed that the most common symptoms were fever (59%), cough (55%), dyspnoea (31%), malaise (30%), fatigue (28%), sore

For peer review only

throat (14%), headache (12%), and chest pain (11%).[14] While it has been demonstrated that PA decreases the risk of severe clinical COVID-19 outcomes (e.g. hospitalisation or death)[15, 16], there is still limited information on the impact of PA on the severity of COVID-19 in patients with less severe disease and on the risk of developing specific symptoms. Therefore, the primary objective of this study was to investigate if the level of PA over the year prior to infection is associated with the severity of the disease in patients positively tested for COVID-19. The secondary objective was to investigate if PA is associated with the most common symptoms: headache, sore throat, fever, dry cough, diarrhoea, breathing difficulties, loss of taste and smell, chest pain, muscle pain, fatigue, confusion and falls. We hypothesised that higher level of PA prior to infection would be associated with less severe forms of COVID-19, as well as with less frequent reports of the major Covid-19 related symptoms.

METHODS

Study design and participants

This is a cross-sectional study using data from a prospective, hybrid cohort study (Predi-COVID) composed of people positively tested for COVID-19 in Luxembourg.[2] The Predi-COVID study aims to identify epidemiological, clinical and sociodemographic characteristics as well as pathogen and/or host predictive biomarkers for the severity of COVID-19. The full study protocol has been published previously [2], with some of the methods that are relevant to this study reproduced below. The study was approved by the National Research Ethics Committee of Luxembourg in April 2020 (study number 202003/07) and registered on www.clinicaltrials.gov (identifier: NCT04380987). All volunteers received a full description of the protocol and provided written informed consent for participation. The findings from this study have been reported according to the *Strengthening the Reporting of Observational Studies in Epidemiology (STROBE)* statement.[17]

All individuals positively tested for COVID-19 in Luxembourg were eligible for the study and contacted by phone by the Health Inspection to enquire whether they consent to having their contact details communicated to the research team. The recruitment took place between Mai 2020 and June 2021. Inclusion

For peer review only - http://bmjopen.bmj.com/site/about/guidelines.xhtml

criteria for this study were: having signed the informed consent, aged above 18 years, confirmed SARS-CoV-2 infection as determined by reverse transcription polymerase chain reaction (RT-PCR), performed by one of the certified laboratories in Luxembourg, and having completed the questionnaire on PA behaviour. Patients already included in another interventional study on COVID-19 and those unable to understand French or German were excluded from the study. The recruitment of participants depended on the emergence and spread of the virus and the resources available.

Patient and public involvement

No patient or public involved.

Outcomes

All clinical data were collected at baseline by research nurses using a modified version of the International Severe Acute Respiratory and Emerging Infection Consortium (ISARIC) case report form. The primary outcome was the severity of illness, which was classified using an adapted version of the National Institute of Health symptom severity classification scheme.[18] Participants were grouped into the following three categories: asymptomatic (positive RT-PCR test and no symptom), mild illness (positive RT-PCR test and one or more symptoms, but no shortness of breath, no symptoms of lower respiratory disease, no abnormal chest imaging) and moderate illness (positive RT-PCR test and symptoms of lower respiratory disease or abnormal chest imaging). The secondary outcomes were specific symptoms reported by the participants at baseline. The presence of the following twelve symptoms was considered for the present work: headache, sore throat, fever, dry cough, diarrhoea, breathing difficulties, loss of taste and smell, chest pain, muscle pain, fatigue, confusion and falls.

Exposures

The exposure was PA over the year prior to infection, which was assessed using a self-reported e-questionnaire using the electronic Patient Reported Outcomes (ePRO) module of Ennov Clinical. The PA

For peer review only - http://bmjopen.bmj.com/site/about/guidelines.xhtml

questionnaire included questions on weekly hours spent walking (to work, shopping, and leisure time), cycling (to work, shopping, and leisure time), gardening (and other handiwork), in household chores, and sports activities (e.g. racket sports, swimming, running) in the year prior to infection, each reported for winter and summer, separately.[19] The time reported for the two seasons was first averaged. Then each activity was assigned a metabolic equivalent task (MET) value based on the Compendium of PA[20], which included MET values of 3.0 for walking and household, 4.0 for gardening and 6.0 for cycling and sports. A total weekly METs score (in MET-h/week) was then calculated from the self-reported data. In addition, PA was categorised into four according to METs score using quartiles.

Covariates

Potential confounders were considered in the analyses and collected with the ISARIC case report form. They included age (years), sex, body mass index (BMI), as well as self-reported comorbidities, smoking status, income and sedentary behaviour. BMI was calculated as measured weight (kg)/height^2 (m^2). Comorbidities included hypertension, chronic heart disease, chronic pulmonary disease, asthma, chronic kidney disease, chronic kidney insufficiency with dialysis, liver disease (mild disease), liver disease (moderate or severe disease), chronic neurological disorders, malignant neoplasia/cancer, chronic hematologic disease, acquired immunodeficiency syndrome, obesity, diabetes with complications, diabetes without complications, rheumatological disease, dementia, malnutrition and chronic obstructive pulmonary disease. As few participants experienced comorbidities, this variable was categorized into "no comorbidity" and "at least one comorbidity". Participants were asked to report whether they are "never smoker", "former smoker" and "current smoker". Income was categorized into "<3000 euro/month", "3000-4999 euro/month", "5000-10000 euro/month" and ">10000 euro/month". Sedentary behaviour was defined as self-reported average number of daily hours spent in sedentary behaviour (e.g. at work, during meal, in front of the screen, etc.) prior to infection.

Statistical analysis

For peer review only - http://bmjopen.bmj.com/site/about/guidelines.xhtml

For peer review only

Descriptive statistics of the study population are presented as counts and percentage for categorical variables and as median and interquartile range (IQR) for not normally distributed continuous variables. Normality was assessed using Shapiro–Wilk test and histograms.

Multiple imputation was performed to deal with missing data. A multivariate imputation by chained equation (MICE) approach was used, assuming a missing at random mechanism. The best predictors were selected based on correlation with the outcomes[21] using the *quickpred* function from the *MICE* package in R. Ten datasets with 20 iterations were imputed and the plausibility of imputations were checked with density plots and summaries. Each imputed dataset was used separately to build the statistical models. Coefficients were pooled and confidence intervals were calculated based on Rubin's rules.[22]

Multinomial logistic regression models were used to investigate the association between PA and illness severity. Two different models were fitted: i) unadjusted model (Model 1), and ii) model 1 adjusted for age, sex, BMI, comorbidities, smoking status, income and sedentary behaviour (Model 2). Separate logistic regression models (fully adjusted) were also used to investigate the association between PA and specific COVID-19 symptoms. For both outcomes, PA was considered as a continuous and a categorical variable in distinct models.

Cubic spline regression models were plotted to investigate the potential non-linear associations between PA and the risk of mild and moderate illness severity, compared to an asymptomatic form, as well as between PA and the risk of specific symptoms. Each cubic spline regression model was defined with four knots, placed at the tertiles of the PA distribution, and with a reference exposure value set at the median of PA for disease severity or a specific symptom, respectively. The *splines* R package was used to fit the models.

All the statistical analyses were performed in R (version 3.6.1) using RStudio (version 1.3.1093). Statistical significance was set to $p<0.05$.

RESULTS

The analysis includes 452 adults, aged [IQR] 42 [31; 51] years old, with confirmed SARS-CoV-2 infection who agreed to participate in the study and provided data on PA. Only five participants were hospitalised, but none

For peer review only

of them was admitted to ICU. Thirteen percent of the participants were asymptomatic (n=59), 63% were classified as mild illness (n=287), and 24% as moderate illness (n=106). The most prevalent symptoms were fatigue (n=294; 65%), headache (n=281; 62%), dry cough (n=241; 53%), muscle pain (n=237; 52%), sore throat (n=203; 45%), fever (n=197; 44%) and loss of taste and smell (n=179; 40%). Breathing difficulties (n=101; 22%), diarrhoea (n=89; 20%), chest pain (n=69; 15%), confusion (n=51; 10%) and falls (n=2; <1%) were less common.

Descriptive statistics of the study population stratified by illness severity are presented in Table 1. Overall, the study population included 48% of women (n=216), median age was 42 (IQR: 31 to 51), BMI was 24.9 (IQR: 22.1 to 27.8), and 79% did not suffer from any comorbidity (n=359). Missing data varied from 0 to 5%. The variables that had missing data were income (n=21; 5%), sedentary behaviour (n=3; 0.66%), BMI (n=2; 0.44%), age (n=1; 0.22%), and smoking status (n=1; 0.22%).

For peer review only

Table 1. Descriptive statistics of the study population stratified by disease severity.

Characteristic	All (n=452)<br>*MED* (*IQR*) or *n* (%)	Disease severity<br>Asymptomatic (n=59)<br>*MED* (*IQR*) or *n* (%)	 Mild illness (n=287)<br>*MED* (*IQR*) or *n* (%)	 Moderate illness (n=106)<br>*MED* (*IQR*) or *n* (%)
Age (years)†	42 (31, 51)	43 (31, 56)	41 (31, 51)	42 (32, 49)
Sex				
Female	216 (47.8)	19 (32.2)	134 (46.7)	63 (59.4)
Male	236 (52.2)	40 (67.8)	153 (53.3)	43 (40.6)
BMI (kg/m^2)†	24.9 (22.1, 27.8)	25.5 (22.2, 28.2)	24.7 (22.1, 27.5)	25.5 (22.2, 29.2)
Comorbidities				
No comorbidities	359 (79.4)	42 (71.2)	243 (84.7)	74 (69.8)
At least one comorbidity	93 (20.6)	17 (28.8)	44 (15.3)	32 (30.2)
Smoking status†				
Never smoker	291 (64.4)	34 (57.6)	184 (64.1)	73 (68.9)
Former smoker	84 (18.6)	13 (22.0)	55 (19.2)	16 (15.1)
Current smoker	77 (17.0)	12 (20.3)	48 (16.7)	17 (16.0)
Income (euro/month)†				
< 3000	71 (15.7)	11 (18.6)	39 (13.6)	21 (19.8)
3000-4999	110 (24.3)	15 (25.4)	70 (24.4)	25 (23.6)
5000-10000	203 (44.9)	23 (39.0)	138 (48.1)	42 (39.6)
> 10000	68 (15.0)	11 (18.6)	40 (13.9)	18 (17.0)
Sedentary behaviour (h/day)†	7 (4, 10)	6 (4, 10)	7 (4, 10)	6 (4, 9)
Physical activity (MET-h/week)	52.9 (30.8, 82.3)	63.0 (40.3, 98.5)	52.00 (31.4, 81.0)	49.3 (27.4, 73.9)
Physical activity (MET-h/week)				
< 30	108 (23.9)	10 (16.9)	68 (23.7)	30 (28.3)
30-52	113 (25.0)	13 (22.0)	75 (26.1)	25 (23.6)
52-82	116 (25.7)	16 (27.1)	74 (25.8)	26 (24.5)
> 82	115 (25.4)	20 (33.9)	70 (24.4)	25 (23.6)

Abbreviations: BMI = body mass index; IQR = interquartile range; MED = median; MET = metabolic equivalent task.
† Imputed data was used for the descriptive statistics.

For peer review only

Table 2 presents the unadjusted and adjusted models for the association between PA and disease severity. When PA was considered as a continuous variable, no association was found with mild or moderate forms of COVID-19 in the unadjusted model. After adjustment, greater PA was associated with a slightly lower risk of moderate illness (odd ratio - OR [95% confidence interval - CI]: 0.99 [0.98; 1.00], *P*=0.041). Cubic spline regression analysis showed that the relationship between PA and the risk of mild or moderate illness was not linear (Figure 1), which supports the use of PA as a categorical variable. The unadjusted model did not reveal any association between PA categories and mild or moderate illness. However, the adjusted model showed a lower risk of moderate illness in the category with the highest PA level (OR [95% CI]: 0.37 [0.14; 0.98], *P*=0.045).

Table 2. Associations between physical activity and illness severity.

Exposure	Outcome	Model 1		Model 2	
		OR (95% CI)	*p*-value	OR (95% CI)	*p*-value
PA (MET-h/week)	Disease severity†				
	Mild illness	0.99 (0.99, 1.00)	0.106	0.99 (0.99, 1.00)	0.064
	Moderate illness	0.99 (0.99, 1.00)	0.068	0.99 (0.98, 1.00)	**0.041***
PA (MET-h/week)††	Disease severity†				
30-52	Mild illness	0.85 (0.35, 2.06)	0.717	0.75 (0.30, 1.88)	0.542
52-82	Mild illness	0.68 (0.29, 1.60)	0.378	0.55 (0.22, 1.34)	0.185
> 82	Mild illness	0.51 (0.22, 1.18)	0.117	0.46 (0.19, 1.08)	0.075
30-52	Moderate illness	0.64 (0.24, 1.71)	0.374	0.57 (0.20, 1.58)	0.278
52-82	Moderate illness	0.54 (0.21, 1.40)	0.205	0.48 (0.18, 1.29)	0.145
> 82	Moderate illness	0.42 (0.16, 1.05)	0.064	0.37 (0.14, 0.98)	**0.045***
p-trend	Mild illness	0.99 (0.98, 1.01)	0.374	0.99 (0.97, 1.01)	0.243
p-trend	Moderate illness	0.99 (0.97, 1.01)	0.203	0.99 (0.97, 1.01)	0.171

Values are presented as OR (95% CI), which were calculated according to Rubin's rule. All models were performed with imputed data. Model 1 = unadjusted model; Model 2 = model 1 adjusted for age, sex, BMI, comorbidities, smoking status, income and sedentary behaviour. Abbreviations: CI = confidence interval; MET = metabolic equivalent task; OR = odds ratio; PA = physical activity.
† Reference: asymptomatic; †† Reference: < 30 MET-h/week; * p-value < 0.05.

Insert Figure 1 about here

For peer review only - http://bmjopen.bmj.com/site/about/guidelines.xhtml

The associations between PA and specific symptoms in the adjusted models are presented in Table 3. Greater PA was associated with lower risk of chest pain (OR [95% CI]: 0.99 [0.98; 1.00], *P*=0.007) when PA was considered as a continuous variable. The category with the highest PA level was associated with lower risk of fatigue (OR [95% CI]: 0.54 [0.30; 0.97], *P*=0.040), dry cough (OR [95% CI]: 0.55 [0.32; 0.96], *P*=0.034), and chest pain (OR [95% CI]: 0.32 [0.14; 0.77], *P*=0.010). Figure 2 shows separate cubic splines investigating the association between PA and specific COVID-19 symptoms.

Table 3. Associations between physical activity and specific COVID-19 symptoms using the adjusted model 2.

**Exposure**	**Outcome**	**OR (95% CI)**	***p*-value**
PA (MET-h/week)	Symptom		
	Fatigue	1.00 (0.99, 1.00)	0.130
	Headache	1.00 (0.99, 1.00)	0.181
	Muscle pain	1.00 (0.99, 1.00)	0.442
	Dry cough	1.00 (0.99, 1.00)	0.056
	Sore throat	1.00 (1.00, 1.00)	0.973
	Fever	1.00 (0.99, 1.00)	0.453
	Loss of taste and smell	1.00 (0.99, 1.00)	0.286
	Breathing difficulties	1.00 (0.99, 1.00)	0.348
	Diarrhoea	1.00 (0.99, 1.00)	0.577
	Chest pain	0.99 (0.98, 1.00)	**0.007***
	Confusion	1.00 (0.99, 1.01)	0.804
PA (MET-h/week)†	Symptom		
30-52		0.63 (0.35, 1.15)	0.130
52-82	Fatigue	0.69 (0.37, 1.25)	0.218
> 82		0.54 (0.30, 0.97)	**0.040***
30-52		1.10 (0.62, 1.94)	0.745
52-82	Headache	1.07 (0.61, 1.89)	0.807
> 82		0.73 (0.42, 1.26)	0.256
30-52		0.80 (0.46, 1.39)	0.430
52-82	Muscle pain	1.15 (0.67, 1.99)	0.615
> 82		0.68 (0.40, 1.18)	0.171
30-52		0.66 (0.38, 1.16)	0.145
52-82	Dry cough	0.69 (0.40, 1.21)	0.196
> 82		0.55 (0.32, 0.96)	**0.034***
30-52		1.24 (0.71, 2.16)	0.453
52-82	Sore throat	1.34 (0.77, 2.33)	0.297
> 82		1.19 (0.69, 2.07)	0.531
30-52		0.97 (0.56, 1.69)	0.924
52-82	Fever	0.90 (0.52, 1.56)	0.700
> 82		0.89 (0.51, 1.54)	0.675
30-52		1.02 (0.58, 1.79)	0.947
52-82	Loss of taste and smell	1.09 (0.62, 1.90)	0.769
> 82		0.84 (0.47, 1.48)	0.539

For peer review only

For peer review only - http://bmjopen.bmj.com/site/about/guidelines.xhtml

30-52	Breathing difficulties	0.64 (0.33, 1.25)	0.190
52-82		0.86 (0.45, 1.62)	0.634
> 82		0.72 (0.38, 1.37)	0.318
30-52	Diarrhoea	0.72 (0.36, 1.43)	0.343
52-82		0.94 (0.49, 1.82)	0.862
> 82		0.70 (0.36, 1.39)	0.313
30-52	Chest pain	0.80 (0.39, 1.65)	0.553
52-82		0.87 (0.43, 1.78)	0.705
> 82		0.32 (0.14, 0.77)	**0.010***
30-52	Confusion	1.04 (0.40, 2.69)	0.941
52-82		2.16 (0.91, 5.09)	0.079
> 82		0.99 (0.38, 2.61)	0.982

Values are presented as OR (95% CI), which were calculated according to Rubin's rule. All models were performed with imputed data. Abbreviations: CI = confidence interval; MET = metabolic equivalent task; OR = odds ratio; PA = physical activity.
† Reference: < 30 MET-h/week; * p-value < 0.05.

Insert Figure 2 about here

DISCUSSION

The protective effect of meeting PA recommendations on the risk of severe COVID-19 outcomes (i.e., death, ICU admission and hospitalisation) has previously been documented.[15, 16] However, whether PA may also prevent less severe illness courses remains unknown. The primary objective of this study was to investigate if the level of PA over the year prior to infection is associated with the severity of the disease in patients positively tested for COVID-19. The secondary objective was to investigate if PA is associated with the most common symptoms such as fatigue, headache, dry cough, muscle pain, sore throat, fever and loss of taste and smell. We hypothesised that higher level of PA prior to the infection would be associated with lower risk of mild and moderate illness, and lower risk of suffering from some of the most commonly reported symptoms. Our main findings were that participants with greater PA were at a lower risk of moderate COVID-19 severity, which confirms our hypothesis. Furthermore, greater level of PA was also associated with a decreased risk of experiencing fatigue, dry cough and chest pain, which are among the most commonly reported symptoms in patients positively tested for COVID-19. These findings suggest that PA is a protective factor for the development of moderate COVID-19 course in adults and for some common related symptoms.

For peer review only - http://bmjopen.bmj.com/site/about/guidelines.xhtml

Previous studies have shown that insufficient PA prior to the pandemic increased the risk of hospitalization[15, 16, 23-25], admission to ICU and death.[15, 16] Notably, low PA was shown to be one of the stronger risk factors for severe COVID-19 outcome, after advanced age and history of organ transplant.[16] Furthermore, meeting the PA guidelines[26] has been shown to decrease the risk of SARS-CoV-2 infection in adult Koreans, beside the negative association with the risk of severe COVID-19 illness (ICU admission or administration of invasive ventilation) and COVID-19 related death.[15] Objective measures of PA have also demonstrated a decreased risk of contracting COVID-19 and hospitalisation in those with greater PA.[27] A study including only patients with chest computed tomography scan confirming infection showed that physical inactivity was associated with the severity of COVID-19 disease.[28] Overall, these previous studies suggested a protective effect of PA for severe COVID-19 outcomes, while some of these studies only included severe cases. While our findings are in line with these previous observations as they confirm the benefits of PA for COVID-19 severity, this is the first study to demonstrate that PA can also provide a protective effect for moderate courses.

Previous scientific literature has supported the role of PA against upper respiratory tract infections.[29] Research on the 2009 H1N1 influenza epidemic demonstrated a dose-response relationship between PA performed before infection and a reduction in the incidence, duration, or severity of acute upper respiratory tract infections.[30] During seasonal influenza, moderately active and active individuals were approximately 15% less likely to visit a physician or emergency services due to influenza compared to inactive individuals.[31] A recent meta-analysis revealed that people engaged in higher levels of PA showed a 31% risk reduction for community acquired infectious disease.[12]

PA can play a protective role against respiratory viral infections and have important roles in a pandemic through three main mechanisms.

For peer review only - http://bmjopen.bmj.com/site/about/guidelines.xhtml

281 First, PA has an indirect protective effect by improving cardiovascular and respiratory functions (i.e. the endurance and strength of the respiratory muscles) and lowering the risk of chronic diseases.[32] Consistently, maximal exercise capacity prior SARS-CoV-2 infection was shown to be inversely associated with the risk of hospitalization due to COVID-19.[33] Exercise capacity is greatly influenced by physical activity, and more specifically regular moderate- to vigorous-intensity aerobic exercise. The authors argued that exercise capacity is an important measure of overall health, the ability of the body to respond to external stressors, and more specifically, the ability to tolerate cardiopulmonary burden.[33]

288 Second, the immune system is very responsive to PA and exercise, with the extent and duration depending on the degree of physiological stress imposed by the workload. Importantly, most of the literature on the effect of PA on human immunity investigated acute effects of exercises and focused on athletes, which call for caution when generalising the findings. Globally, the beneficial effect of regular PA on the immune system may involve several mechanisms such as enhanced immunosurveillance, reduced systemic inflammation and improved regulation of the immune system as well as delayed onset of immunosenescence.[34, 35] A recent systematic review investigated the effects of regular PA on the immune system.[12] Interventions including 3–5 sessions per week for an average of 30 min at moderate to vigorous intensity (e.g., walking, running, cycling) resulted overall in a lower concentration of neutrophils, as well as a higher concentrations of CD4 T helper cells and salivary immunoglobulin (IgA). The lower concentration of neutrophil may be interpreted as a consequence of the beneficial effect of regular physical activity on chronic inflammation.[36] CD4 T cells contribute to a rapid and more robust immune response. Salivary IgA can be regarded as the first line of defence of the immune system on the mucosal surface and plays other roles such as down-regulating inflammation processes.[37] Among others, experimental studies have also showed that moderate intensity PA stimulates an increase in the antipathogen activity of immune system macrophages and anti-inflammatory cytokines in the blood, together resulting in a reduced influx of inflammatory cells into the lungs.[29]

305 Third, PA may also enhance vaccination response [38, 39] and has a direct impact on trained immunity of innate immune cells such as Kupffer cells in the liver.[40] Trained immunity on the other hand, which

For peer review only

describes a long-term boost through metabolic and epigenetic reprogramming of the innate immune response by certain stimuli (such as BCG vaccination or PA), has been proposed as an important tool for reducing susceptibility to and severity of COVID-19.[41]

Some studies have described a “J” shaped association between exercise volume and infection with optimal protection at moderate levels of activity.[42] In this study, the cubic spline plots showed that the relationships between PA and COVID-19 severity and symptoms occurrence were not linear, but our sample size did not enable to define the shape of the curve accurately.

One of the limitations of this study was the use of self-reported measures to assess PA, which might have resulted in recall bias, compared with exposure assessment measured using objective means (i.e., accelerometers), which can provide a more accurate assessment of the true level of PA. However, our PA assessment tool has previously been used in large cohort studies[19, 43], and covers all the PA domains (i.e., occupational, transportation, leisure-time, household/gardening). Nevertheless, the use of self-report questionnaires usually leads to overestimation of PA, which may lead to underestimation of the magnitude of true associations.[44] Second, there was no measure of PA intensity, although each activity was assigned a specific MET value. Third, this study was an observational study with a limited sample size for some outcome categories. It is not possible to conclude that PA prior to infection is causally related to less severe COVID-19 outcomes as this study design suffers from a potential issue of residual confounding due to unmeasured or unknown confounders. However, our adjusted model controlled for all the most relevant confounders identified so far. Fourth, some estimated 95% CI suggest sparse data bias (see Table 2 and 3), which should be recognised as an important limitation.

CONCLUSION

We found that greater PA prior to infection was associated with a reduced risk of moderate illness severity among adults positively tested for COVID-19. Greater PA was also associated with a reduced risk of

For peer review only

experiencing fatigue, dry cough and chest pain, which are among the most commonly reported symptoms in patients with COVID-19. This study provides new evidence that PA is a modifiable risk factor for COVID-19 severity, including moderate illness. Our findings suggest that engaging in regular PA may be one of the key actions individuals can take to minimise adverse consequences of COVID-19.

AUTHOR CONTRIBUTIONS

LM, AB, AF, GA, MO, GF contributed sufficiently to the manuscript to justify authorship. LM, AF, GA, MO and GF conceptualised the project, and LM, AB, GA and GF defined the methodology for the present study. LM and AB verified the underlying data and conducted the data analysis. All authors were involved in the interpretation of the analysis results. LM drafted the first manuscript and all other authors provided significant feedback and comments to refine the final manuscript. All authors approved the final manuscript and confirm that they accept responsibility to submit for publication.

ACKNOWLEDGEMENTS

We thank the Predi-COVID participants for their involvement in the study, the members of the Predi-COVID external scientific committee for their expertise, as well as the project team, the IT team in charge of the app development and the nurses in charge of recruitment, data and sample collection and management on the field.

FUNDING/SUPPORT

The Predi-COVID study is supported by the Luxembourg National Research Fund (FNR) (Predi-COVID, grant number 14716273), the André Losch Fondation and the Luxembourg Institute of Health. The study funders had no role in the design and conduct of the study; collection, management, analysis, and interpretation of the data; preparation, review, or approval of the manuscript; or decision to submit the manuscript for publication.

For peer review only

COMPETING INTERESTS

The authors did not have any competing interest.

DATA SHARING STATEMENT

As this is a cross-sectional analysis of baseline data from a currently ongoing prospective study, the data will not be made available publicly before the end of the Predi-Covid study. The study protocol can be found under https://bmjopen.bmj.com/content/10/11/e041834.abstract.

ETHICS STATEMENT

The study was approved by the National Research Ethics Committee of Luxembourg (CNER) in April 2020 (ID: 202003/07).

For peer review only

REFERENCES

1. Liu YC, Kuo RL, Shih SR. COVID-19: The first documented coronavirus pandemic in history. *Biomed J* 2020;43(4):328-33.

2. Fagherazzi G, Fischer A, Betsou F, et al. Protocol for a prospective, longitudinal cohort of people with COVID-19 and their household members to study factors associated with disease severity: The Predi-COVID study. *BMJ Open* 2020;10(11):e041834.

3. Izcovich A, Ragusa MA, Tortosa F, et al. Prognostic factors for severity and mortality in patients infected with COVID-19: A systematic review. *PloS one* 2020;15(11):e0241955.

4. Bastard P, Gervais A, Le Voyer T, et al. Autoantibodies neutralizing type I IFNs are present in~ 4% of uninfected individuals over 70 years old and account for~ 20% of COVID-19 deaths. *Science Immunology* 2021;6(62):eabl4340.

5. Asano T, Boisson B, Onodi F, et al. X-linked recessive TLR7 deficiency in~ 1% of men under 60 years old with life-threatening COVID-19. *Science Immunology* 2021;6(62):eabl4348.

6. Jordan RE, Adab P, Cheng K. Covid-19: Risk factors for severe disease and death. British Medical Journal Publishing Group; 2020.

7. Lee IM, Shiroma EJ, Lobelo F, et al. Effect of physical inactivity on major non-communicable diseases worldwide: An analysis of burden of disease and life expectancy. *Lancet* 2012;380(9838):219-29.

8. Lacombe J, Armstrong MEG, Wright FL, et al. The impact of physical activity and an additional behavioural risk factor on cardiovascular disease, cancer and all-cause mortality: A systematic review. *BMC Public Health* 2019;19(1):900.

9. Wahid A, Manek N, Nichols M, et al. Quantifying the association between physical activity and cardiovascular disease and diabetes: A systematic review and meta-analysis. *J Am Heart Assoc* 2016;5(9):e002495.

10. Bialek S, Boundy E, Bowen V, et al. Severe outcomes among patients with coronavirus disease 2019 (COVID-19)—United States, February 12–March 16, 2020. *Morb Mortal Wkly Rep* 2020;69(12):343.

11. Lavie CJ, Ozemek C, Carbone S, et al. Sedentary behavior, exercise, and cardiovascular health. *Circ Res* 2019;124(5):799-815.

12. Chastin SF, Abaraogu U, Bourgois JG, et al. Effects of regular physical activity on the immune system, vaccination and risk of community-acquired infectious disease in the general population: Systematic review and meta-analysis. *Sports Med* 2021:1-14.

13. Yuki K, Fujiogi M, Koutsogiannaki S. COVID-19 pathophysiology: A review. *Clin Immunol* 2020;215:108427.

14. da Rosa Mesquita R, Francelino Silva Junior LC, Santos Santana FM, et al. Clinical manifestations of COVID-19 in the general population: Systematic review. *Wien Klin Wochenschr* 2021;133(7-8):377-82.

15. Lee SW, Lee J, Moon SY, et al. Physical activity and the risk of SARS-CoV-2 infection, severe COVID-19 illness and COVID-19 related mortality in South Korea: A nationwide cohort study. *Br J Sports Med* 2021.

16. Sallis R, Young DR, Tartof SY, et al. Physical inactivity is associated with a higher risk for severe COVID-19 outcomes: A study in 48 440 adult patients. *Br J Sports Med* 2021.

17. von Elm E, Altman DG, Egger M, et al. The Strengthening the Reporting of Observational Studies in Epidemiology (STROBE) statement: Guidelines for reporting observational studies. *PLoS Med* 2007;4(10):e296.

For peer review only - http://bmjopen.bmj.com/site/about/guidelines.xhtml

18. National Institutes of Health (NIH). Clinical spectrum of SARS-CoV-2 infection 2021. https://www.covid19treatmentguidelines.nih.gov/overview/clinical-spectrum/ (accessed July 23, 2021).

19. MacDonald CJ, Madika AL, Lajous M, et al. Associations between physical activity and incident hypertension across strata of body mass index: A prospective investigation in a large cohort of french women. *J Am Heart Assoc* 2020;9(23):e015121.

20. Ainsworth BE, Haskell WL, Whitt MC, et al. Compendium of physical activities: an update of activity codes and MET intensities. *Med Sci Sports Exerc* 2000;32(9 Suppl):S498-504.

21. Van Buuren S, Groothuis-Oudshoorn K. mice: Multivariate imputation by chained equations in R. *J Stat Softw* 2011;45(1):1-67.

22. Rubin DB. Multiple imputation for nonresponse in surveys: John Wiley & Sons 2004.

23. Hamer M, Kivimaki M, Gale CR, et al. Lifestyle risk factors, inflammatory mechanisms, and COVID-19 hospitalization: A community-based cohort study of 387,109 adults in UK. *Brain Behav Immun* 2020;87:184-87.

24. Yuan Q, Huang H-y, Chen X-l, et al. Does pre-existent physical inactivity have a role in the severity of COVID-19? *Ther Adv Respir Dis* 2021;15:17534666211025221.

25. de Souza FR, Motta-Santos D, Dos Santos Soares D, et al. Association of physical activity levels and the prevalence of COVID-19-associated hospitalization. *J Sci Med Sport* 2021;24(9):913-18.

26. Piercy KL, Troiano RP, Ballard RM, et al. The Physical Activity Guidelines for Americans. *JAMA* 2018;320(19):2020-28.

27. Zhang X, Li X, Sun Z, et al. Physical activity and COVID-19: An observational and Mendelian randomisation study. *J Glob Health* 2020;10(2):020514.

28. Tavakol Z, Ghannadi S, Tabesh MR, et al. Relationship between physical activity, healthy lifestyle and COVID-19 disease severity: A cross-sectional study. *J Public Health* 2021:1-9.

29. Laddu DR, Lavie CJ, Phillips SA, et al. Physical activity for immunity protection: Inoculating populations with healthy living medicine in preparation for the next pandemic. *Prog Cardiovasc Dis* 2021;64:102-04.

30. Wong CM, Lai HK, Ou CQ, et al. Is exercise protective against influenza-associated mortality? *PLoS One* 2008;3(5):e2108.

31. Siu E, Campitelli MA, Kwong JC. Physical activity and influenza-coded outpatient visits: A population-based cohort study. *PLoS One* 2012;7(6):e39518.

32. Nieman DC. Coronavirus disease-2019: A tocsin to our aging, unfit, corpulent, and immunodeficient society. *J Sport Health Sci* 2020;9(4):293-301.

33. Brawner CA, Ehrman JK, Bole S, et al., editors. Inverse relationship of maximal exercise capacity to hospitalization secondary to coronavirus disease 2019. Mayo Clinic Proceedings; 2021: Elsevier.

34. Simpson RJ, Kunz H, Agha N, et al. Exercise and the regulation of immune functions. *Prog Mol Biol Transl Sci* 2015;135:355-80.

35. Nieman DC, Wentz LM. The compelling link between physical activity and the body's defense system. *J Sport Health Sci* 2019;8(3):201-17.

36. Hamer M, Sabia S, Batty GD, et al. Physical activity and inflammatory markers over 10 years: follow-up in men and women from the Whitehall II cohort study. *Circulation* 2012;126(8):928-33.

37. Monteiro R. Immunoglobulin A as an anti-inflammatory agent. *Clinical and experimental immunology* 2014;178(Suppl 1):108.

For peer review only

38. Pascoe AR, Fiatarone Singh MA, Edwards KM. The effects of exercise on vaccination responses: A review of chronic and acute exercise interventions in humans. *Brain Behav Immun* 2014;39:33-41.

39. Gualano B, Lemes IR, da Silva RP, et al. Association between physical activity and immunogenicity of an inactivated virus vaccine against SARS-CoV-2 in patients with autoimmune rheumatic diseases. *Brain, behavior, and immunity* 2021.

40. Zhang H, Chen T, Ren J, et al. Pre-operative exercise therapy triggers anti-inflammatory trained immunity of Kupffer cells through metabolic reprogramming. *Nature metabolism* 2021;3(6):843-58.

41. Netea MG, Giamarellos-Bourboulis EJ, Domínguez-Andrés J, et al. Trained immunity: a tool for reducing susceptibility to and the severity of SARS-CoV-2 infection. *Cell* 2020;181(5):969-77.

42. Schwellnus M, Soligard T, Alonso J-M, et al. How much is too much?(Part 2) International Olympic Committee consensus statement on load in sport and risk of illness. *Br J Sports Med* 2016;50(17):1043-52.

43. Fournier A, Dos Santos G, Guillas G, et al. Recent recreational physical activity and breast cancer risk in postmenopausal women in the E3N cohort. *Cancer Epidemiol Biomarkers Prev* 2014;23(9):1893-902.

44. Stamatakis E, Lee I-M, Bennie J, et al. Does strength-promoting exercise confer unique health benefits? A pooled analysis of data on 11 population cohorts with all-cause, cancer, and cardiovascular mortality endpoints. *Am J Epidemiol* 2018;187(5):1102-12.

For peer review only

FIGURES

Figure 1.

Figure title: Cubic spline regression investigating the association between physical activity and disease severity. Reference exposure value set at the median of physical activity (52.9 MET-h/week).

Figure 2.

Figure title: Cubic spline regression investigating the association between physical activity and specific COVID-19 symptoms. Reference exposure value set at the median of physical activity (52.9 MET-h/week).

For peer review only

For peer review only - http://bmjopen.bmj.com/site/about/guidelines.xhtml

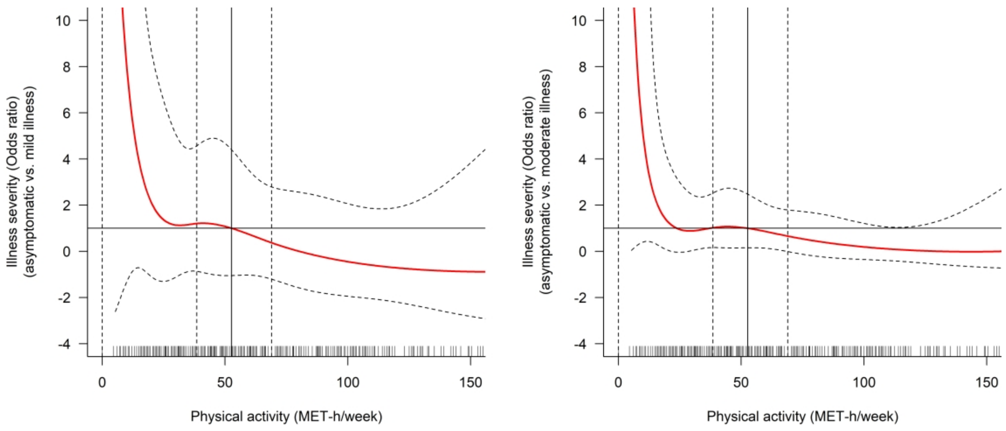

Cubic spline regression investigating the association between physical activity and disease severity. Reference exposure value set at the median of physical activity (52.9 MET-h/week).

762x317mm (236 x 236 DPI)

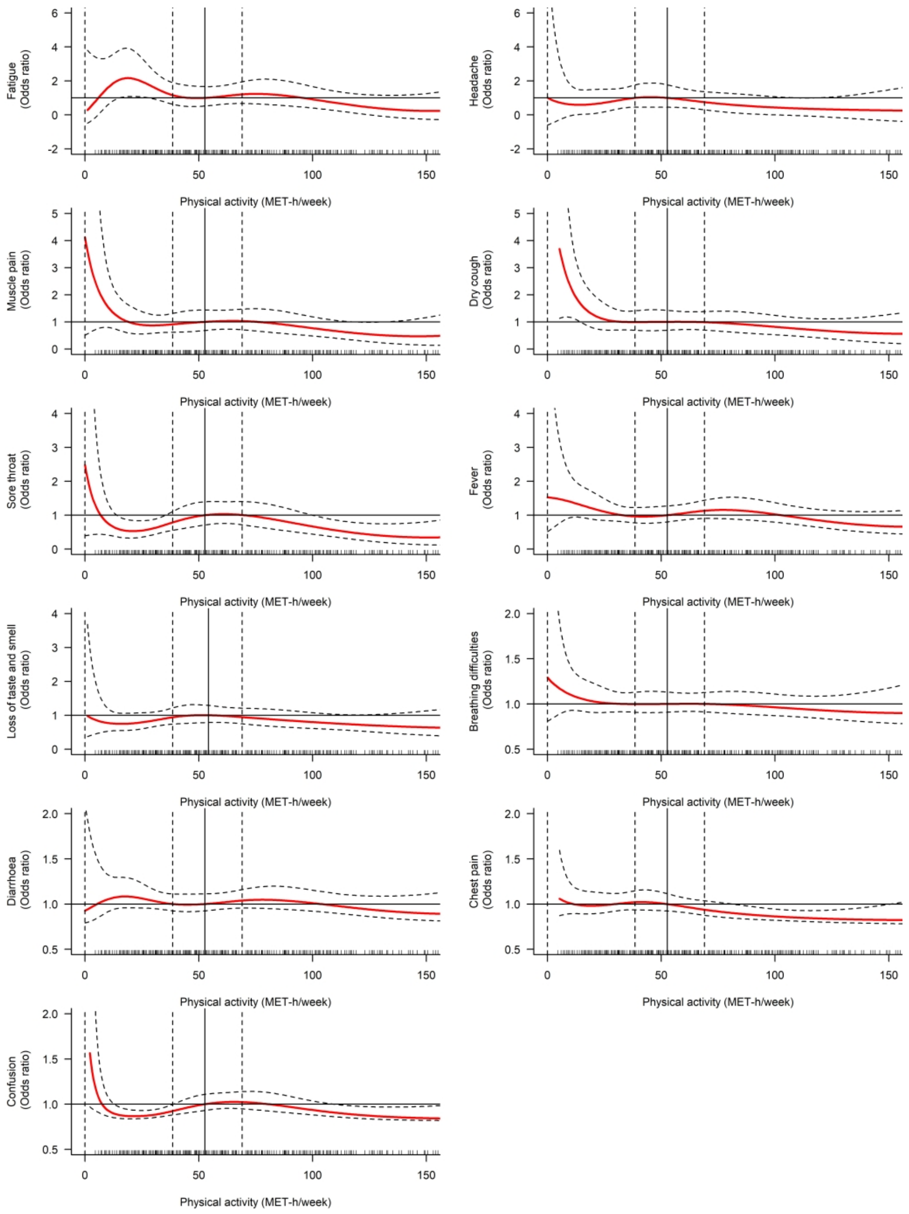

Cubic spline regression investigating the association between physical activity and specific COVID-19 symptoms. Reference exposure value set at the median of physical activity (52.9 MET-h/week).

571x762mm (236 x 236 DPI)

STROBE Statement—Checklist of items that should be included in reports of ***cross-sectional studies***

	Item No	Recommendation	Pages
**Title and abstract**	1	(*a*) Indicate the study's design with a commonly used term in the title or the abstract	2
		(*b*) Provide in the abstract an informative and balanced summary of what was done and what was found	2
**Introduction**			
Background/rationale	2	Explain the scientific background and rationale for the investigation being reported	4-5
Objectives	3	State specific objectives, including any prespecified hypotheses	5
**Methods**			
Study design	4	Present key elements of study design early in the paper	5
Setting	5	Describe the setting, locations, and relevant dates, including periods of recruitment, exposure, follow-up, and data collection	5-6
Participants	6	(*a*) Give the eligibility criteria, and the sources and methods of selection of participants	5-6
Variables	7	Clearly define all outcomes, exposures, predictors, potential confounders, and effect modifiers. Give diagnostic criteria, if applicable	6-7
Data sources/ measurement	8*	For each variable of interest, give sources of data and details of methods of assessment (measurement). Describe comparability of assessment methods if there is more than one group	6-7
Bias	9	Describe any efforts to address potential sources of bias	7-8
Study size	10	Explain how the study size was arrived at	5 and 8
Quantitative variables	11	Explain how quantitative variables were handled in the analyses. If applicable, describe which groupings were chosen and why	6-7
Statistical methods	12	(*a*) Describe all statistical methods, including those used to control for confounding	7-8
		(*b*) Describe any methods used to examine subgroups and interactions	n.a.
		(*c*) Explain how missing data were addressed	7-8
		(*d*) If applicable, describe analytical methods taking account of sampling strategy	n.a.
		(*e*) Describe any sensitivity analyses	n.a.
**Results**			
Participants	13*	(a) Report numbers of individuals at each stage of study—eg numbers potentially eligible, examined for eligibility, confirmed eligible, included in the study, completing follow-up, and analysed	8-9
		(b) Give reasons for non-participation at each stage	n.a.
		(c) Consider use of a flow diagram	n.a.

For peer review only - http://bmjopen.bmj.com/site/about/guidelines.xhtml

Descriptive data	14*	(a) Give characteristics of study participants (eg demographic, clinical, social) and information on exposures and potential confounders	8-9 (table 1)
		(b) Indicate number of participants with missing data for each variable of interest	9
Outcome data	15*	Report numbers of outcome events or summary measures	8-9
Main results	16	(*a*) Give unadjusted estimates and, if applicable, confounder-adjusted estimates and their precision (eg, 95% confidence interval). Make clear which confounders were adjusted for and why they were included	11-12 (tables 2 & 3)
		(*b*) Report category boundaries when continuous variables were categorized	7 (tables 2 & 3)
		(*c*) If relevant, consider translating estimates of relative risk into absolute risk for a meaningful time period	n.a.
Other analyses	17	Report other analyses done—eg analyses of subgroups and interactions, and sensitivity analyses	n.a.
**Discussion**			
Key results	18	Summarise key results with reference to study objectives	13
Limitations	19	Discuss limitations of the study, taking into account sources of potential bias or imprecision. Discuss both direction and magnitude of any potential bias	15-16
Interpretation	20	Give a cautious overall interpretation of results considering objectives, limitations, multiplicity of analyses, results from similar studies, and other relevant evidence	14-15
Generalisability	21	Discuss the generalisability (external validity) of the study results	13-14, 16
**Other information**			
Funding	22	Give the source of funding and the role of the funders for the present study and, if applicable, for the original study on which the present article is based	17

*Give information separately for exposed and unexposed groups.

Note: An Explanation and Elaboration article discusses each checklist item and gives methodological background and published examples of transparent reporting. The STROBE checklist is best used in conjunction with this article (freely available on the Web sites of PLoS Medicine at http://www.plosmedicine.org/, Annals of Internal Medicine at http://www.annals.org/, and Epidemiology at http://www.epidem.com/). Information on the STROBE Initiative is available at www.strobe-statement.org.

For peer review only
